# Supplementary figures and images for: PIGB maintains nuclear lamina organization in skeletal muscle of Drosophila
Source: J Cell Biol. 2024 Jan 23;223(2):e202301062. doi: 10.1083/jcb.202301062 (PMC10808031; doi:10.1083/jcb.202301062)

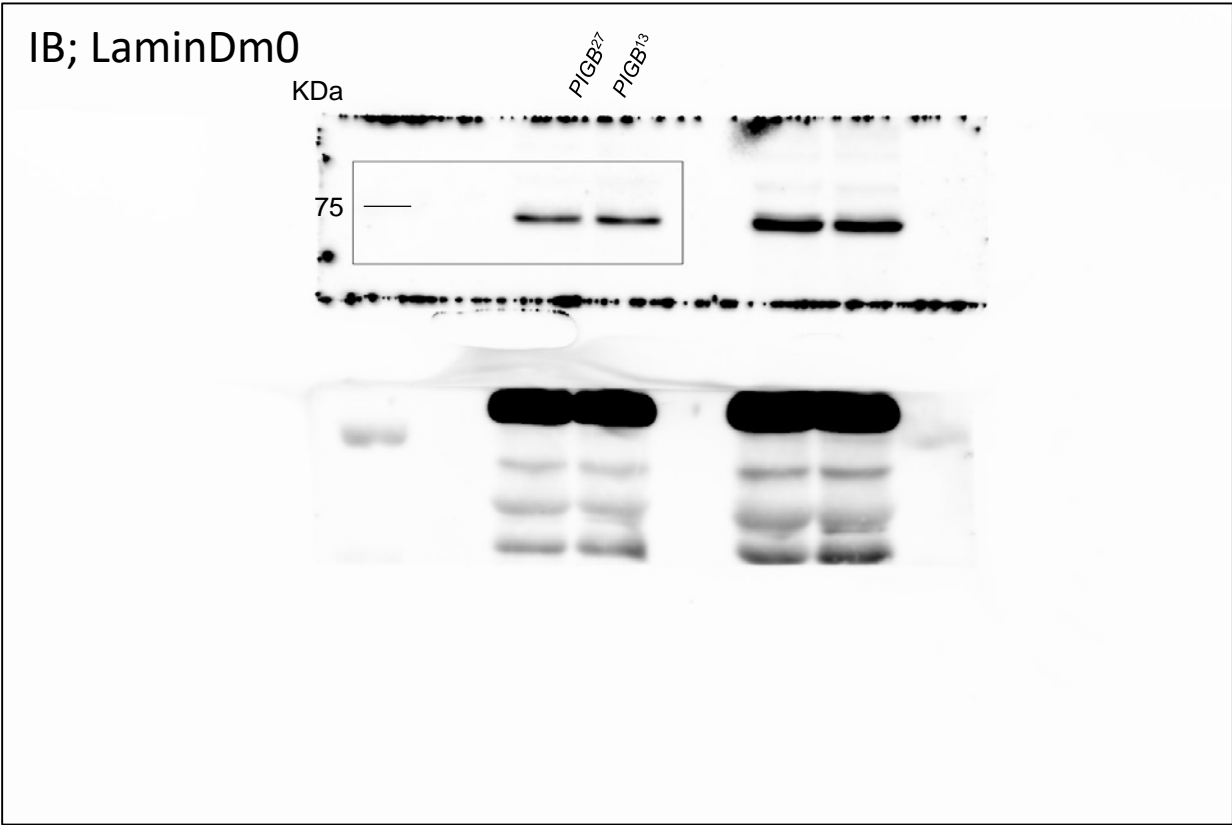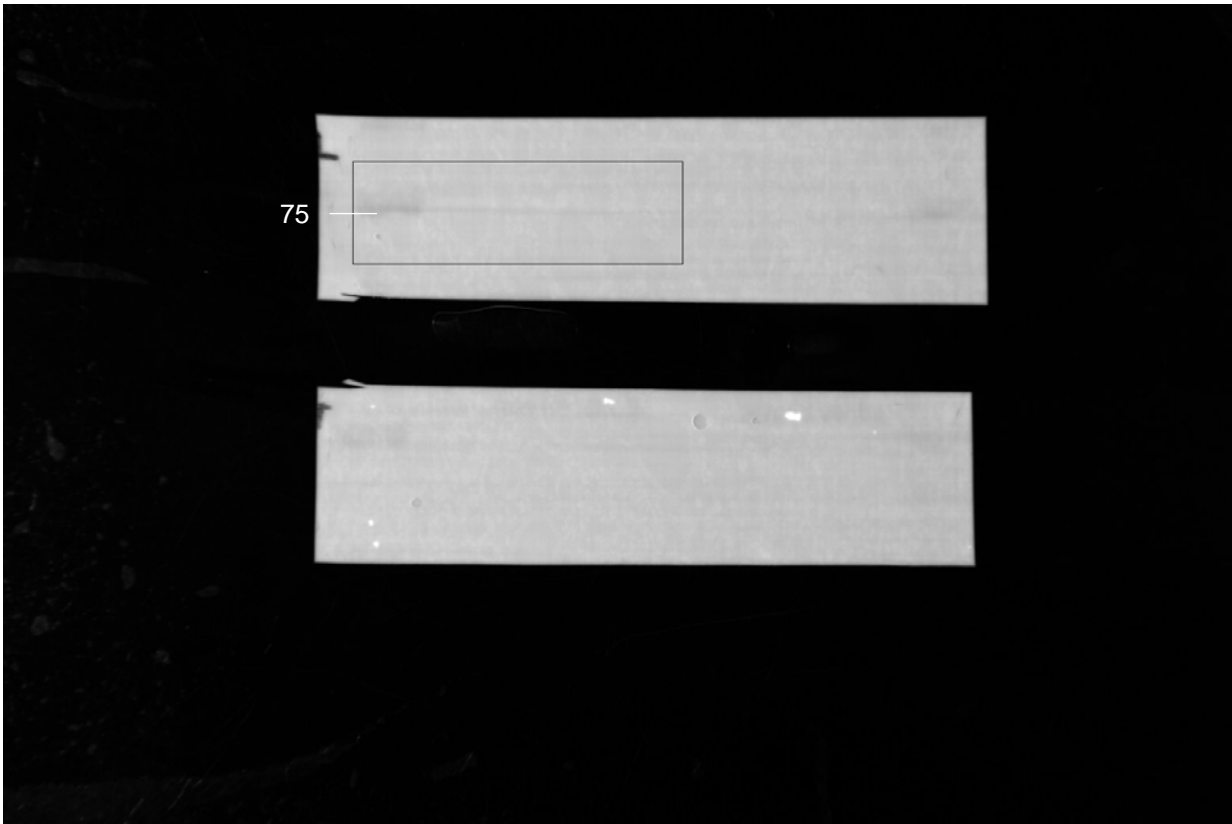

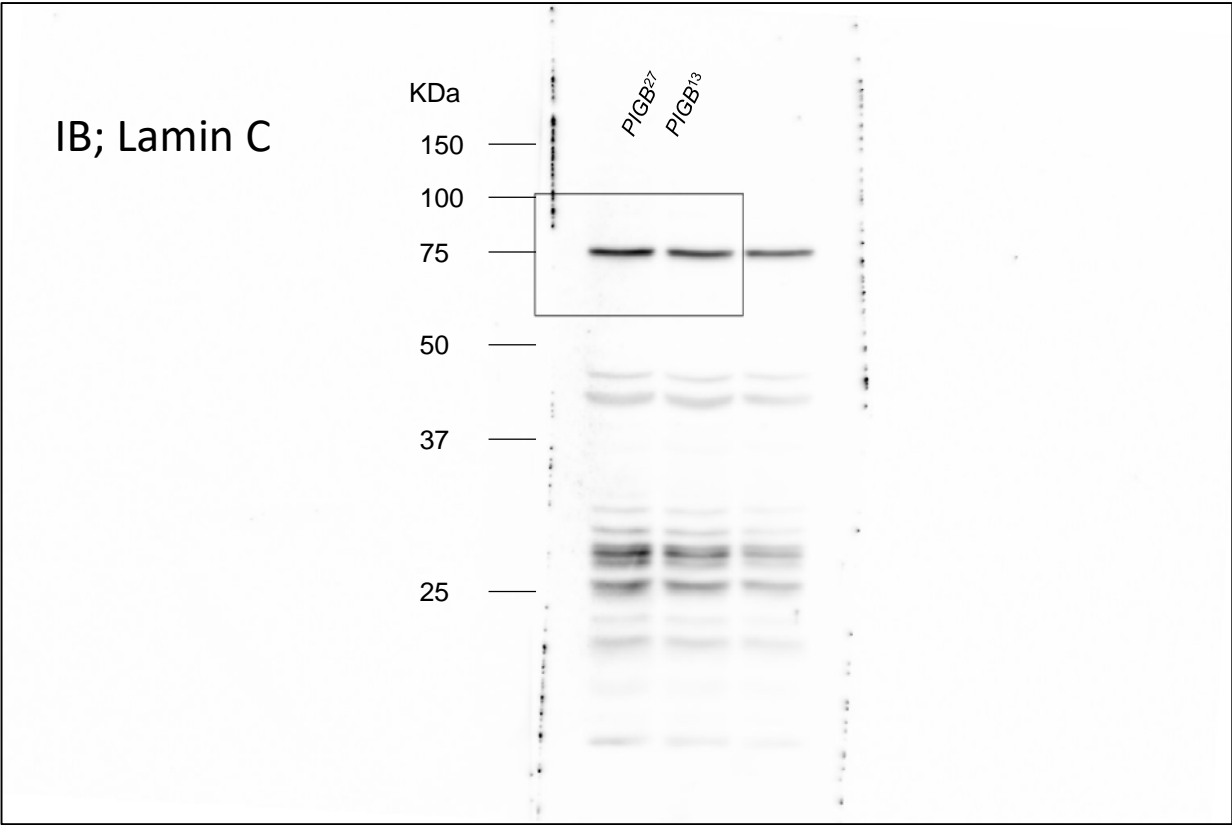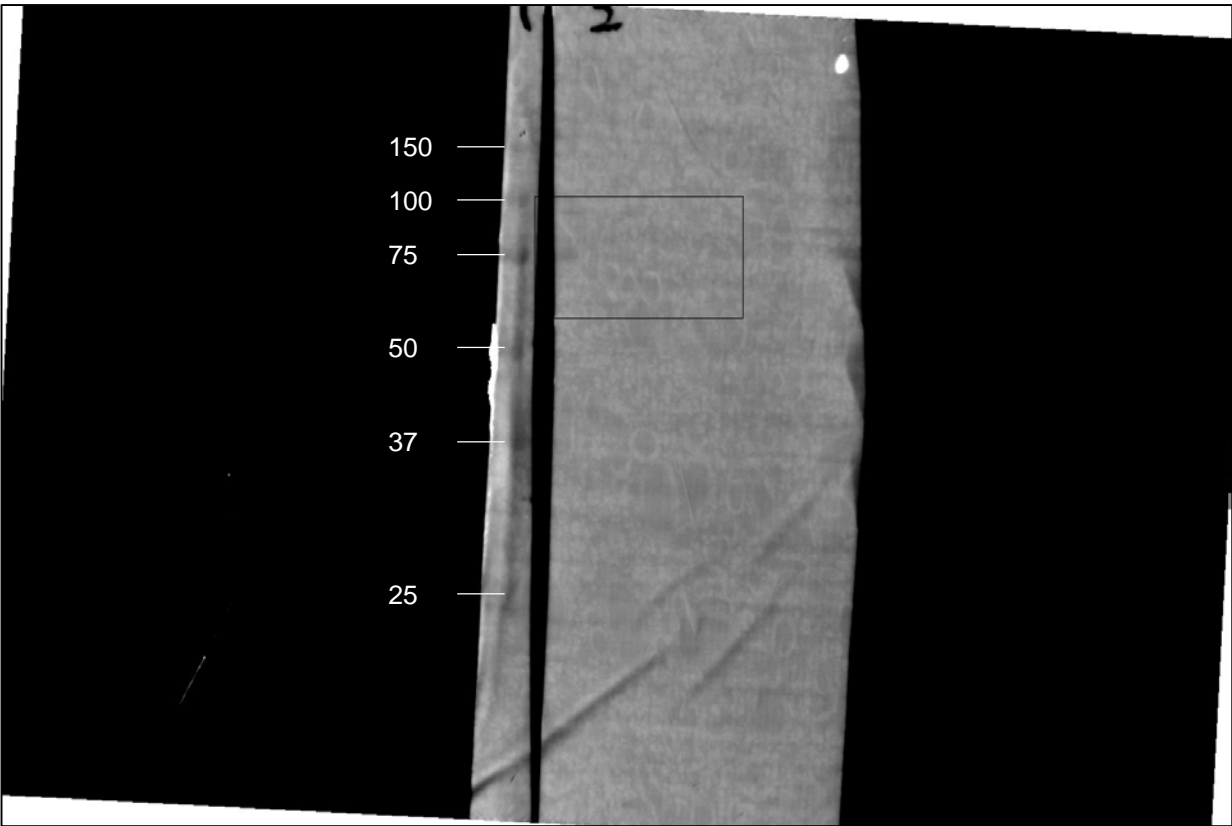

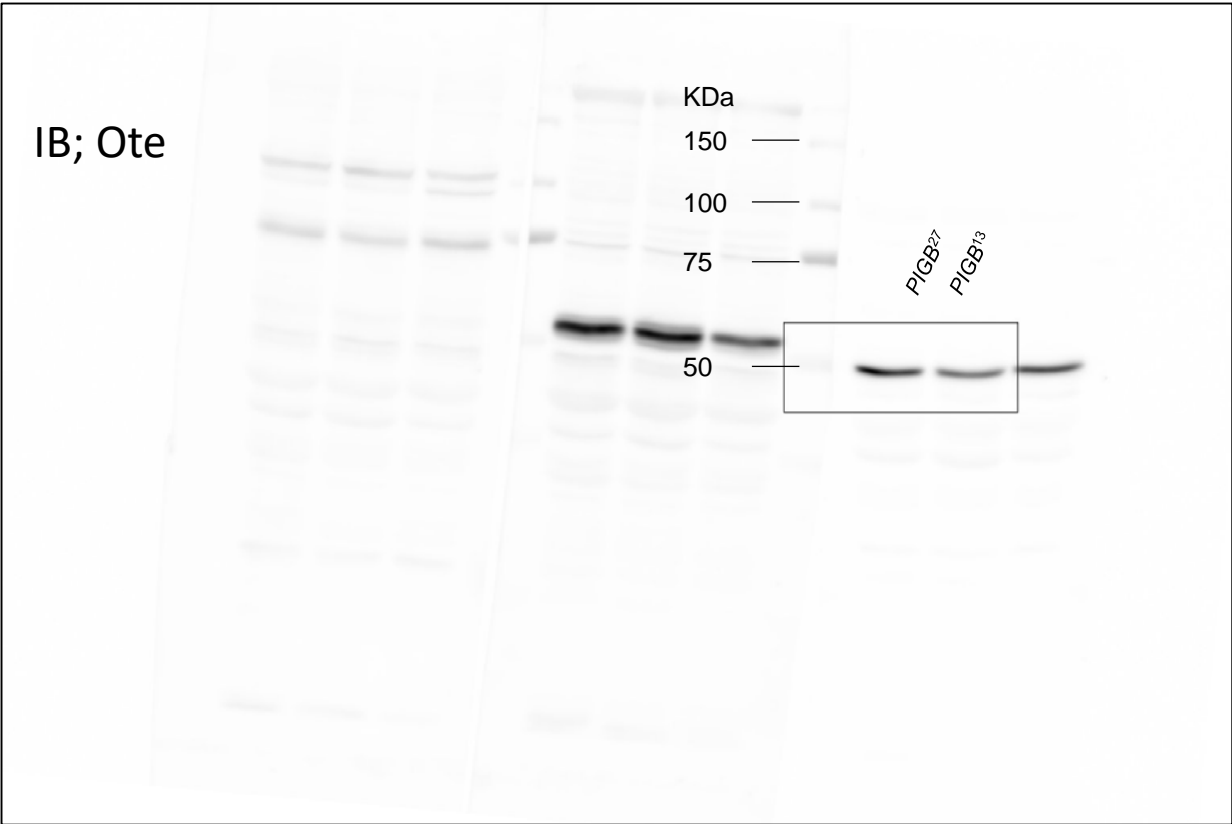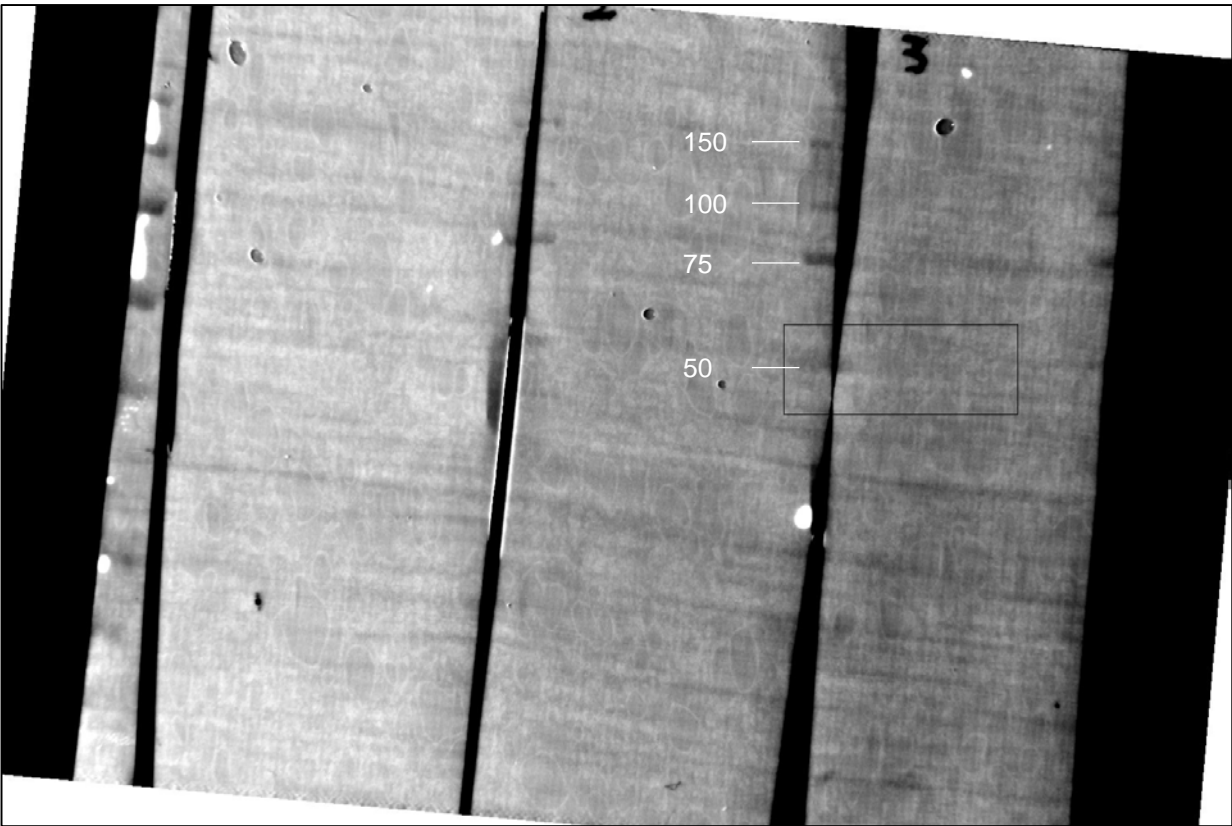

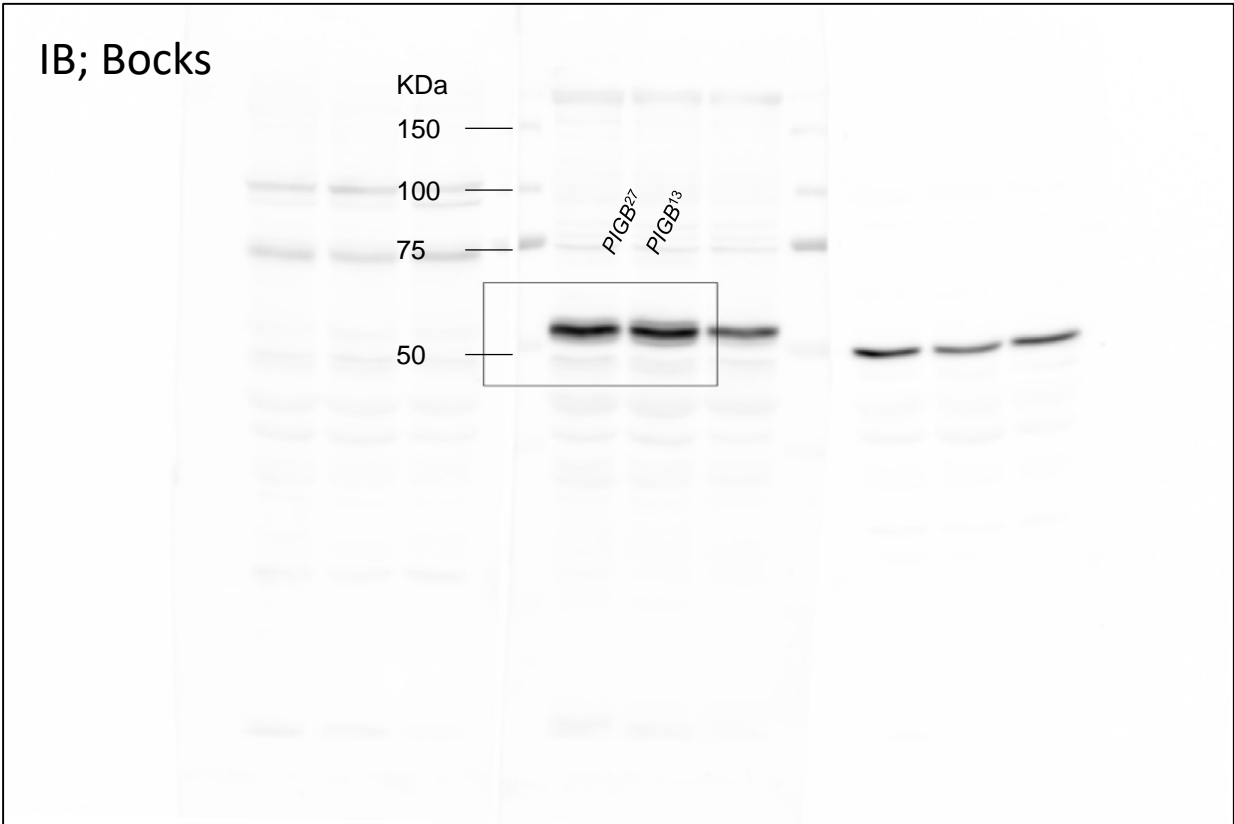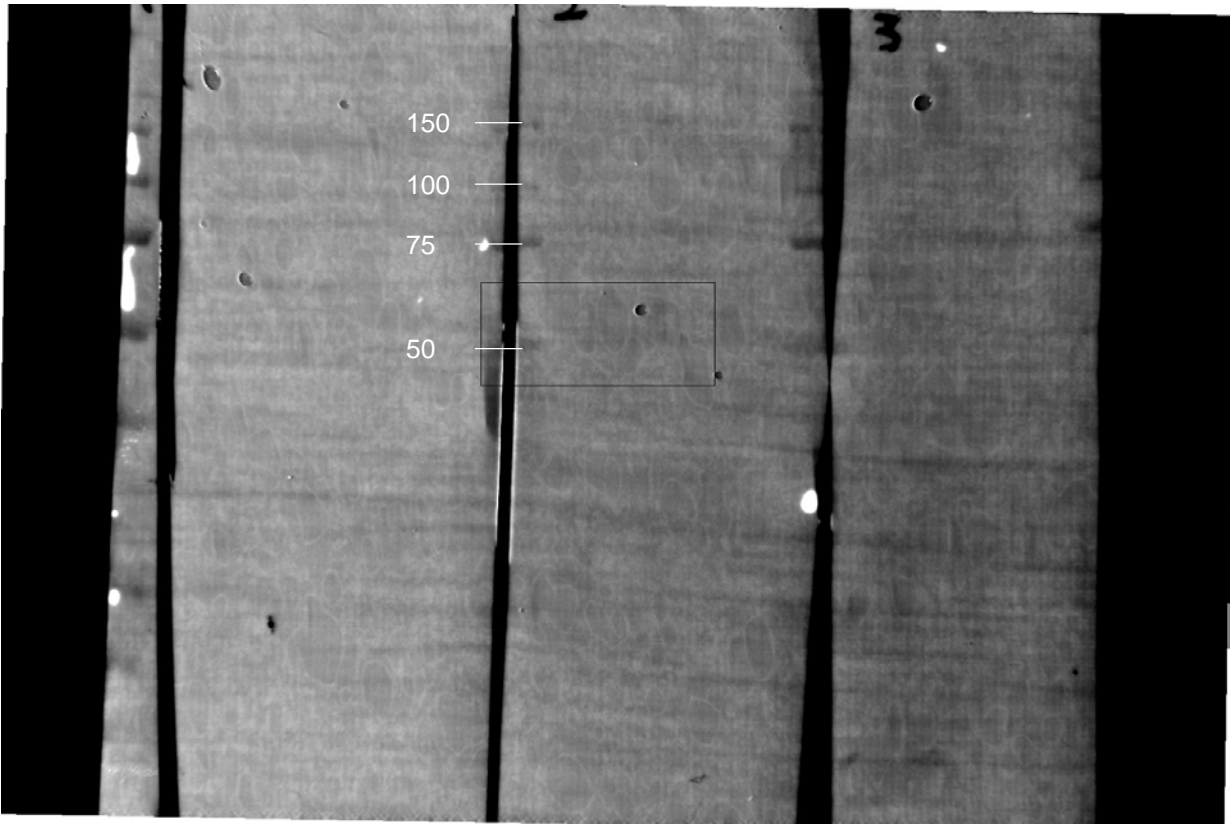

IB; LBR

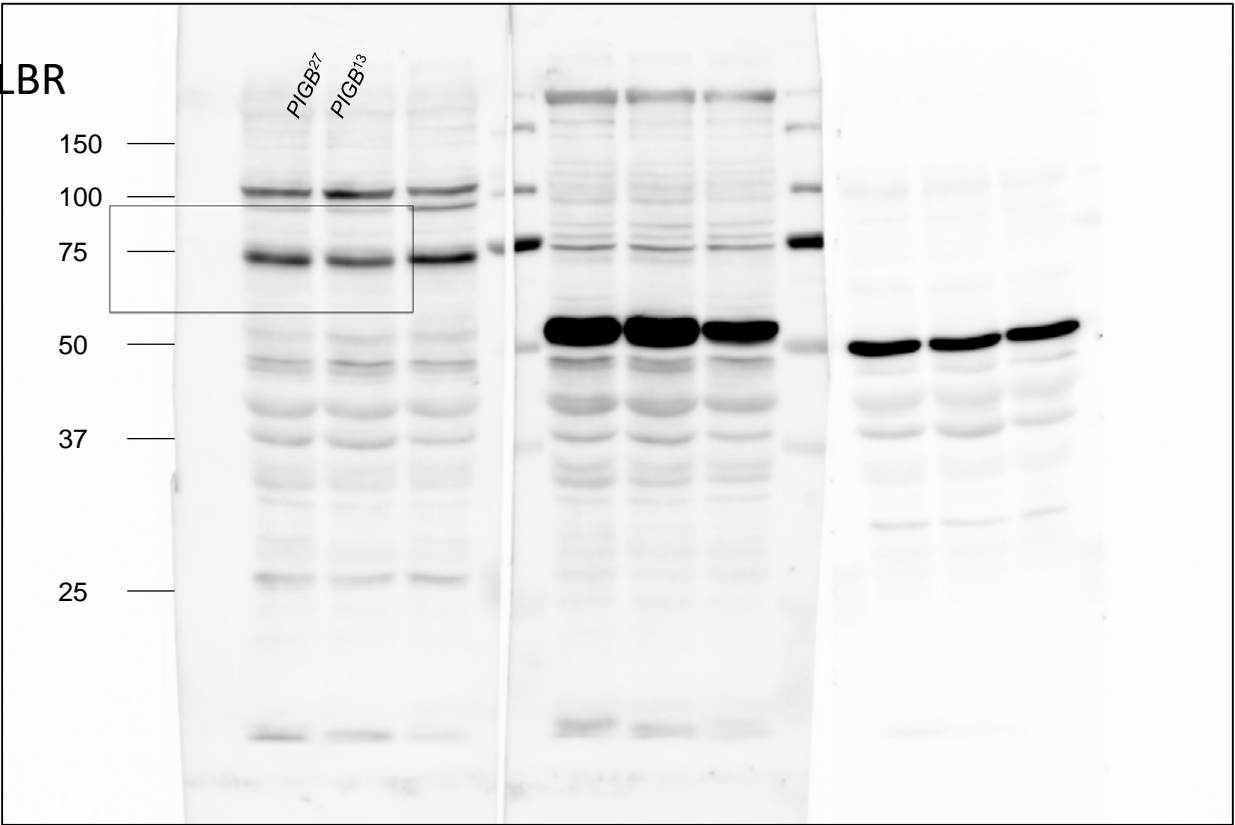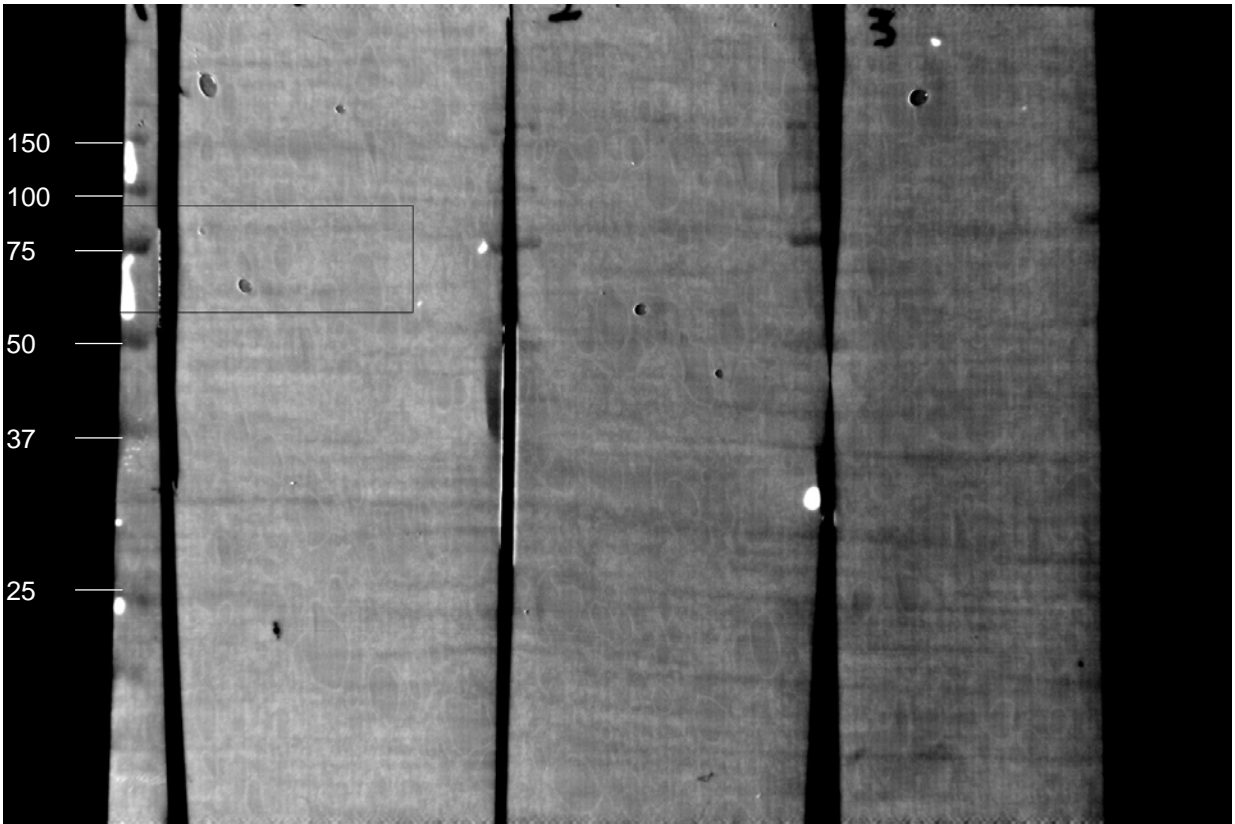

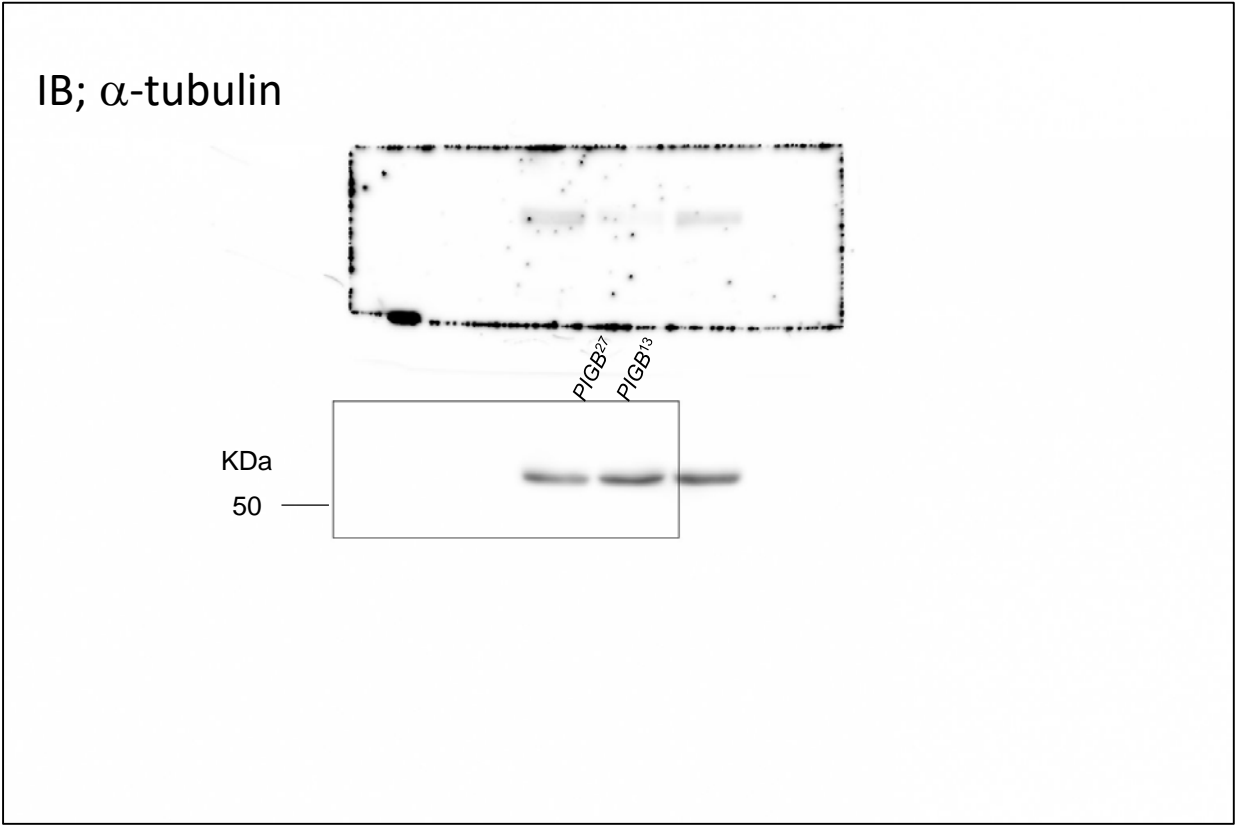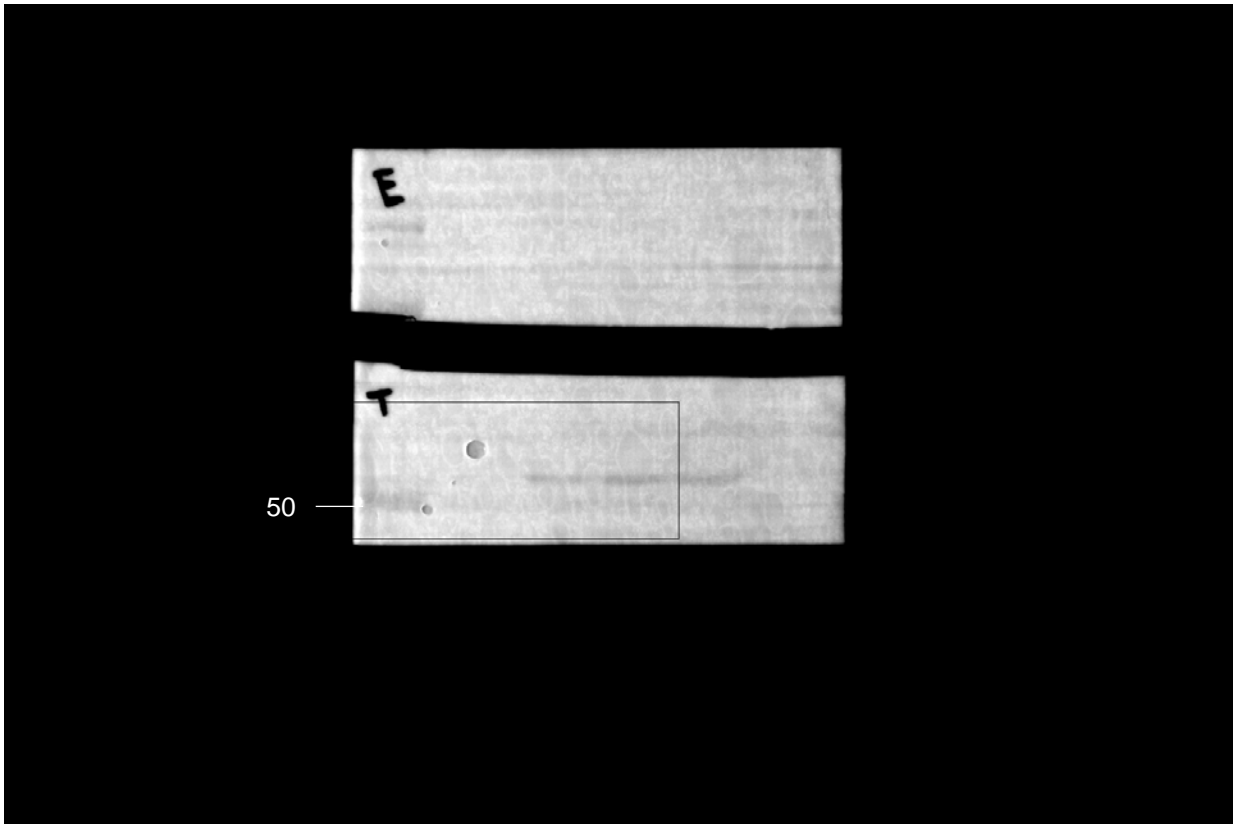

IB; LaminDm0

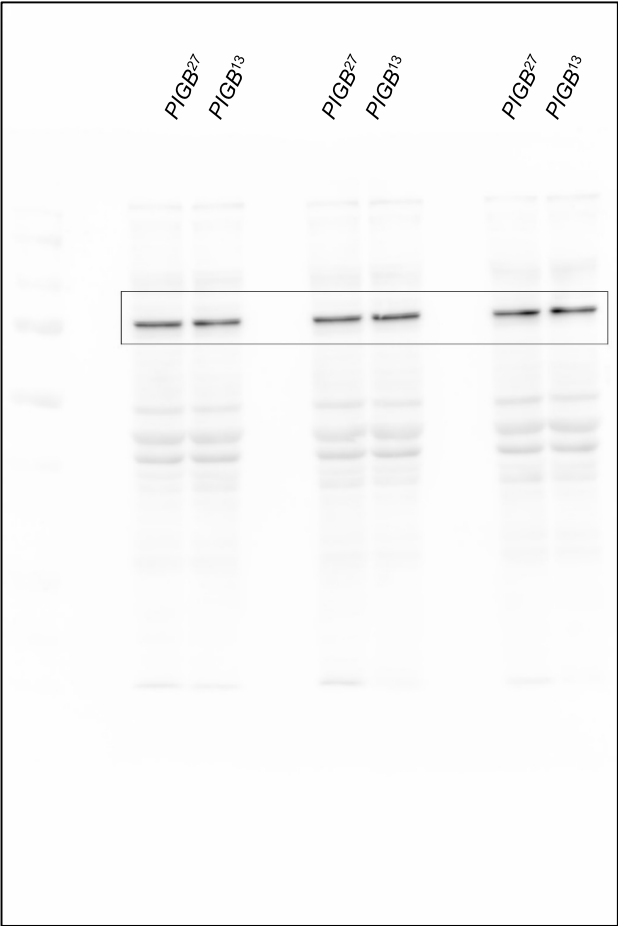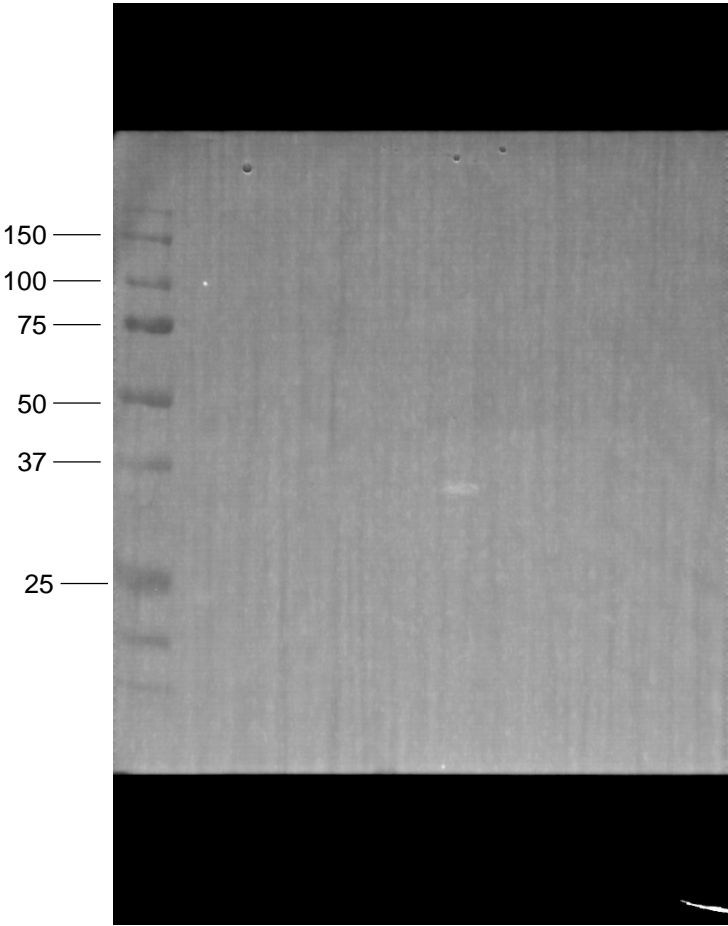

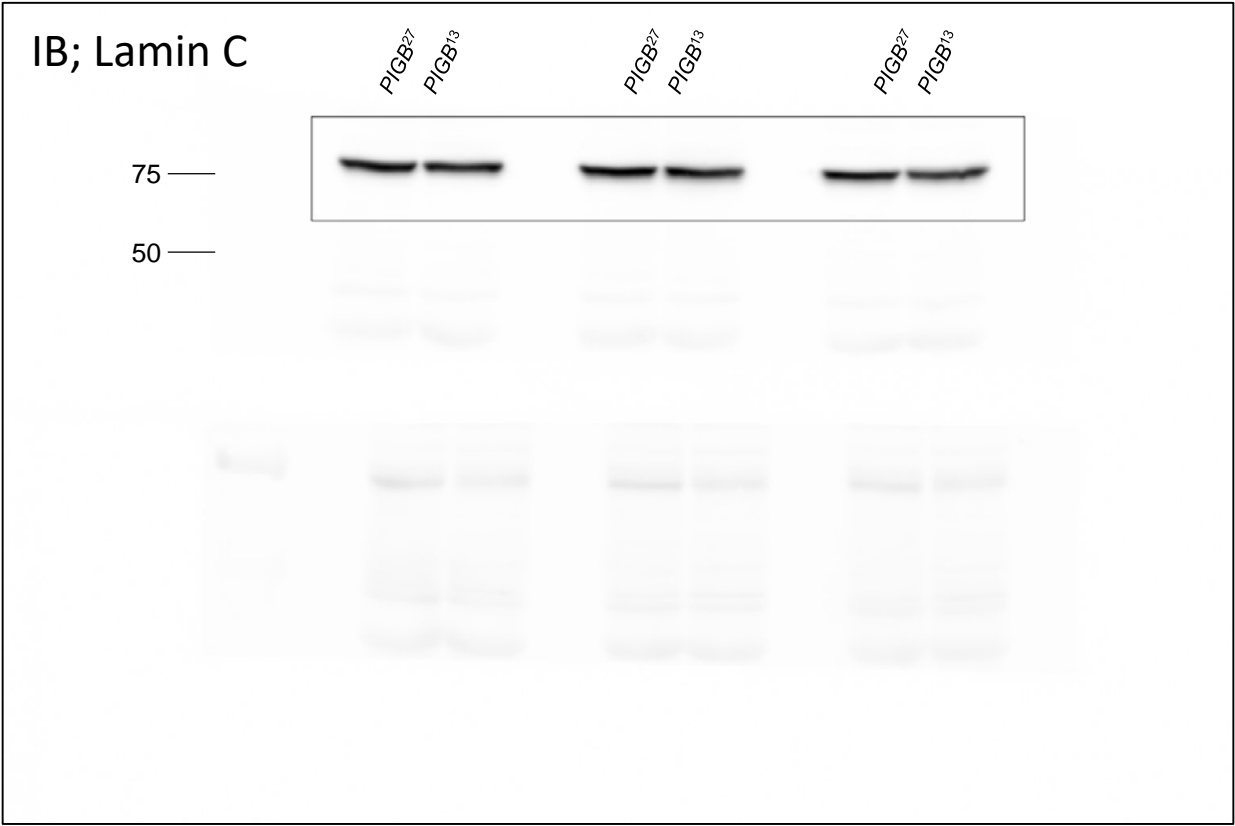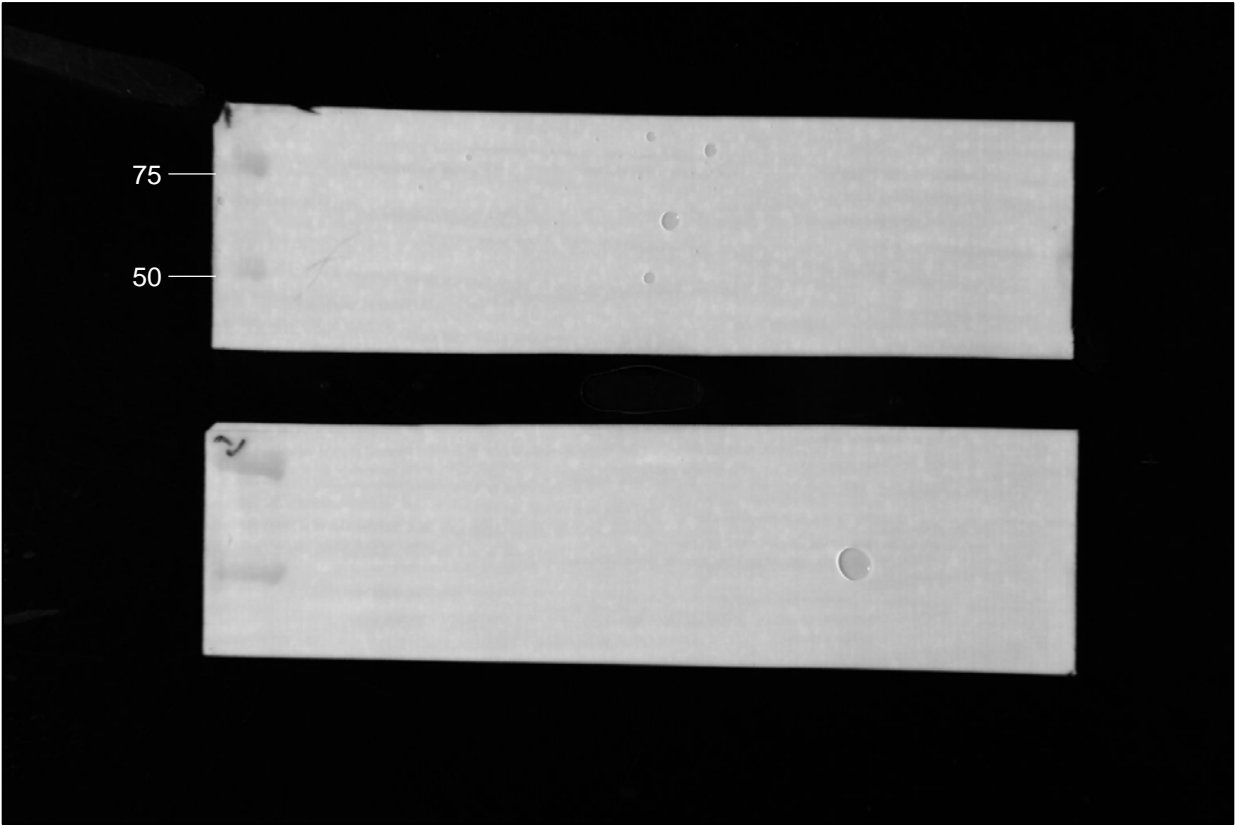

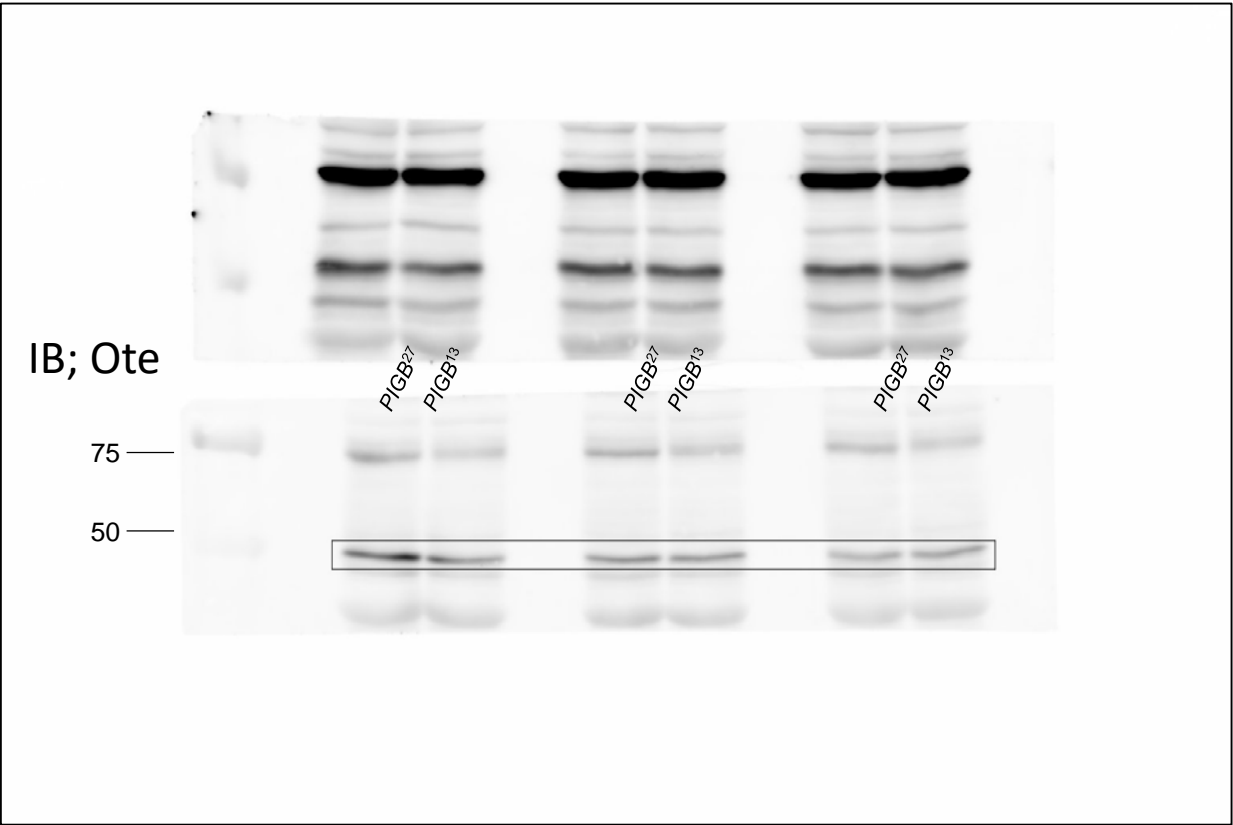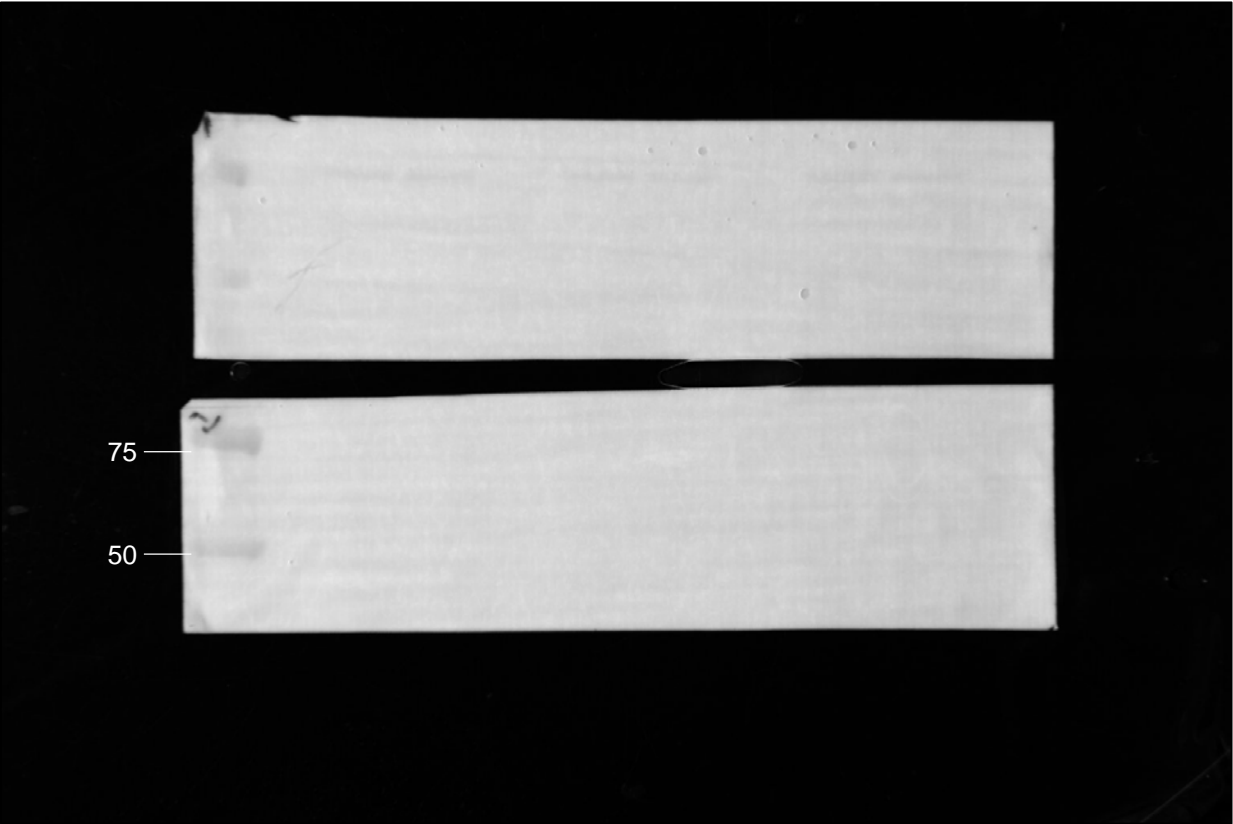

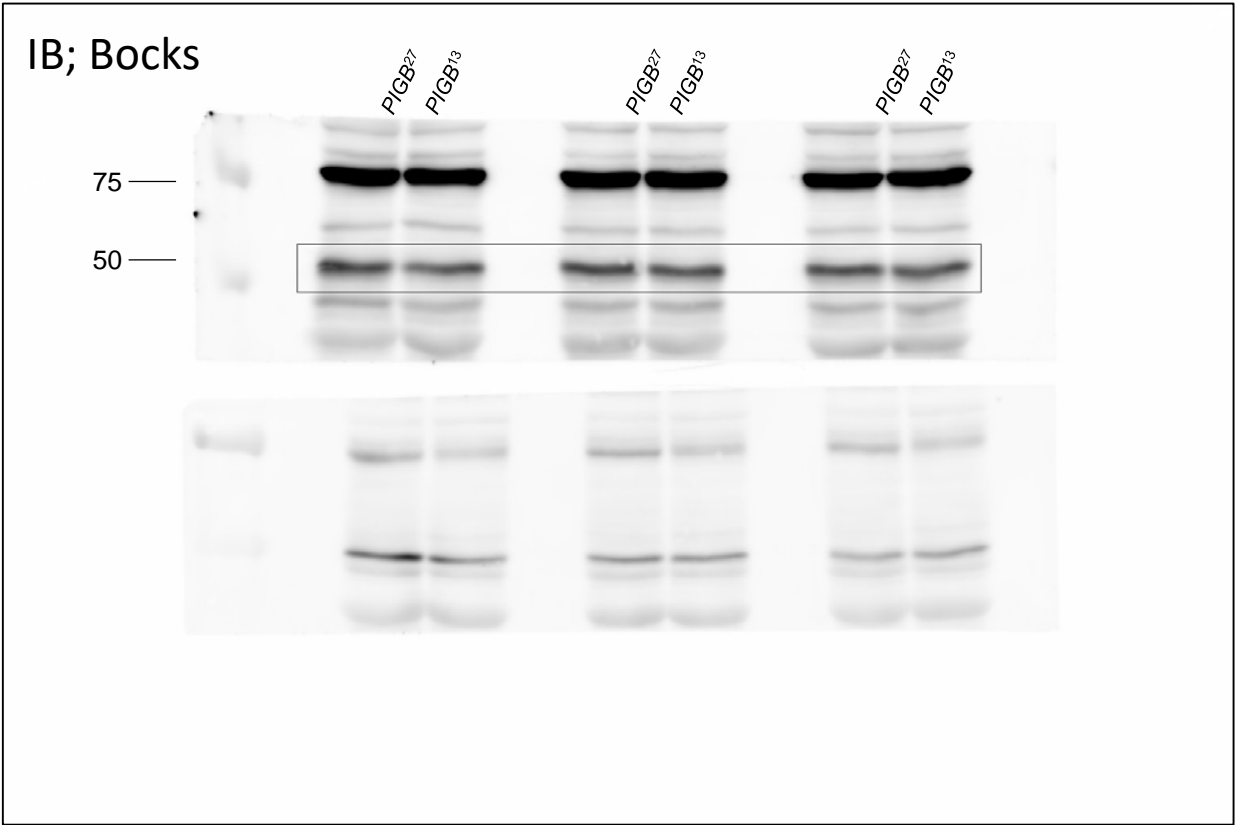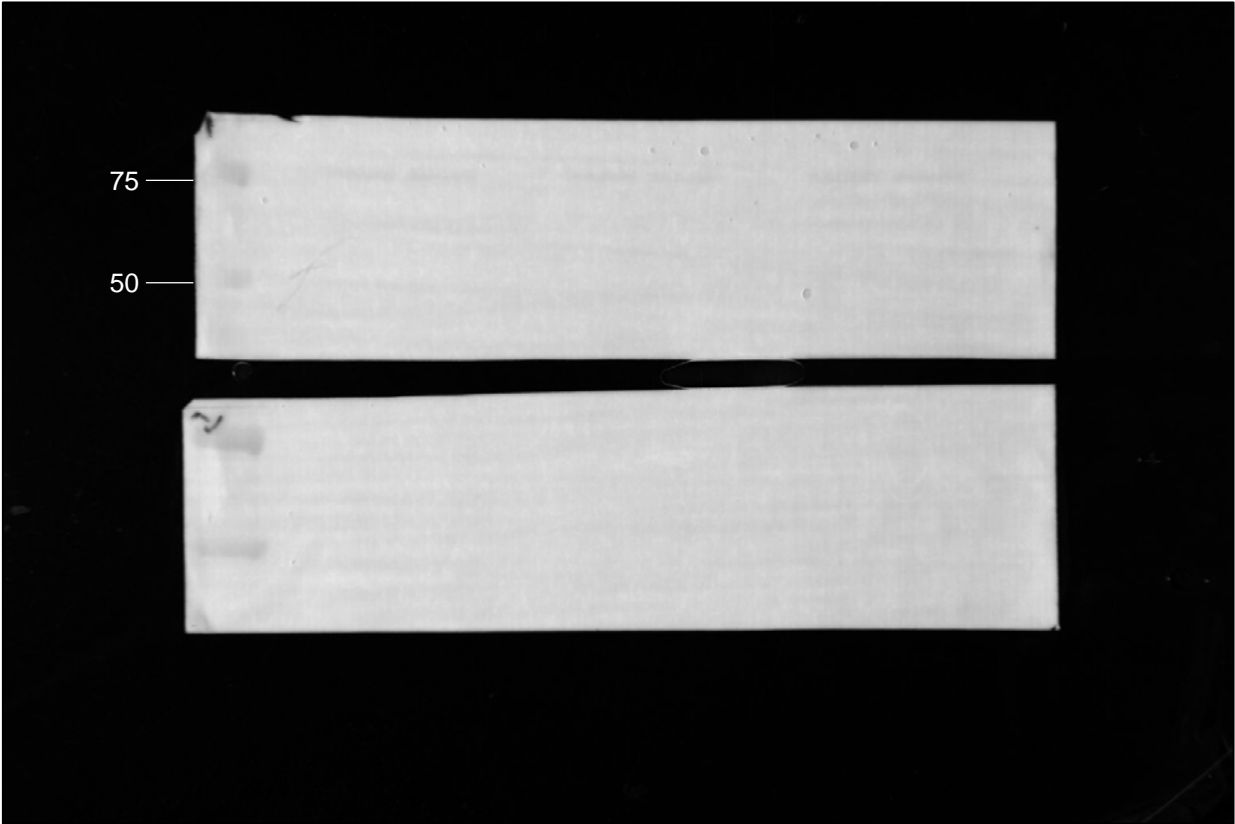

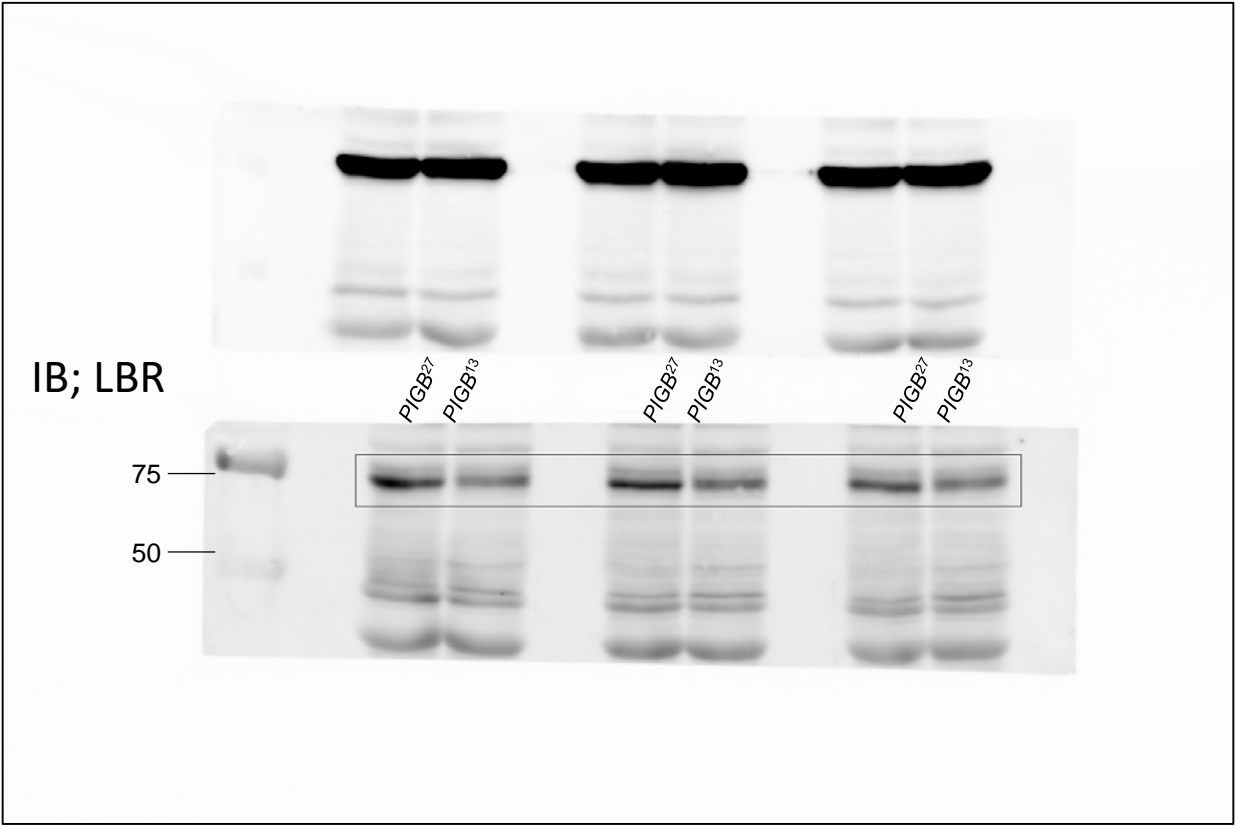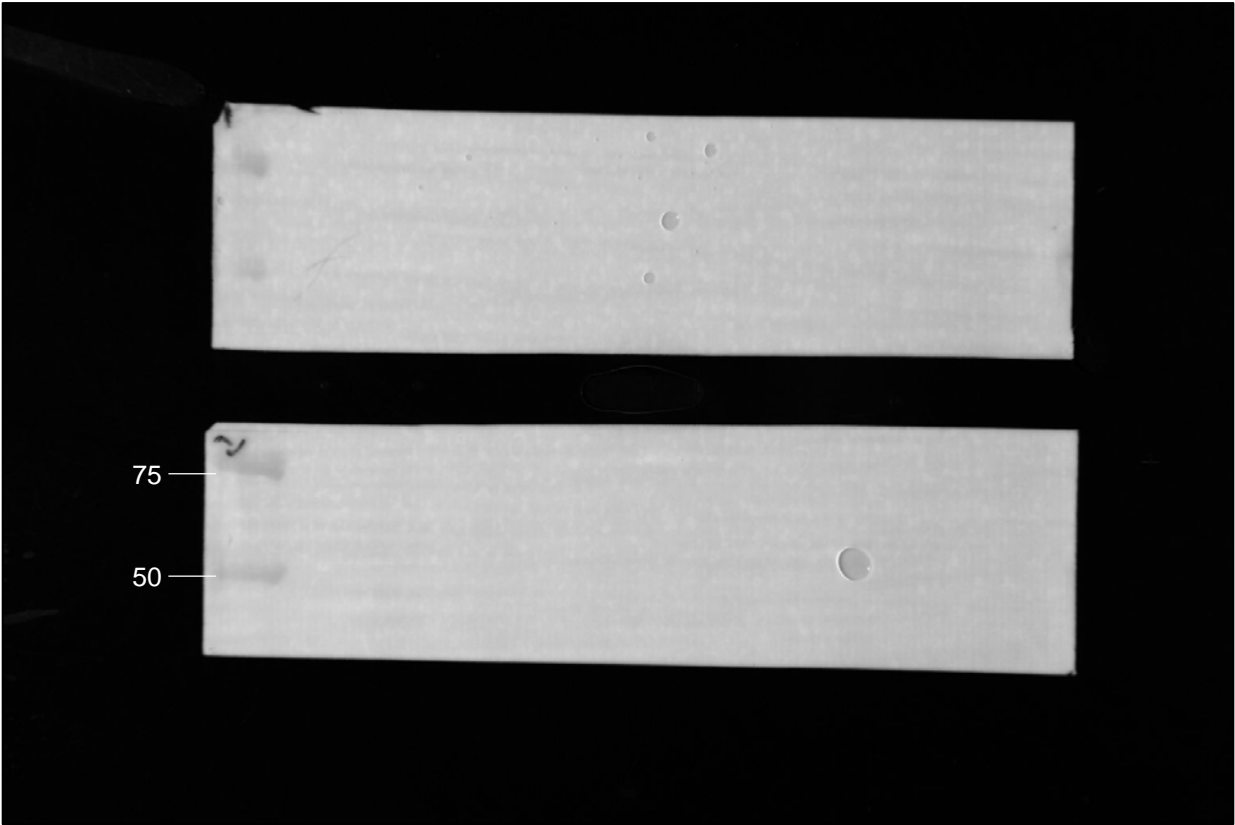

IB;  $\alpha$ -tubulin

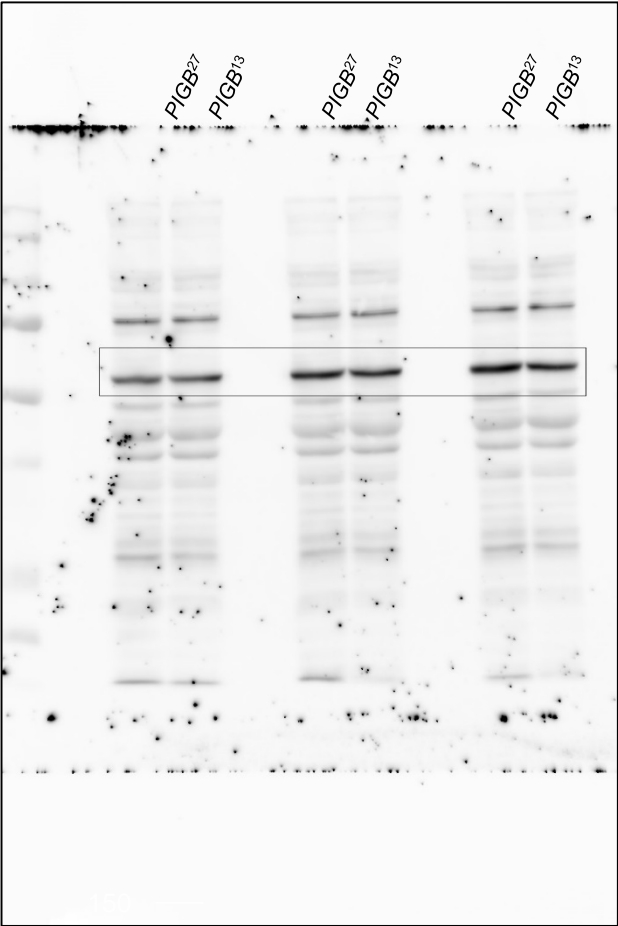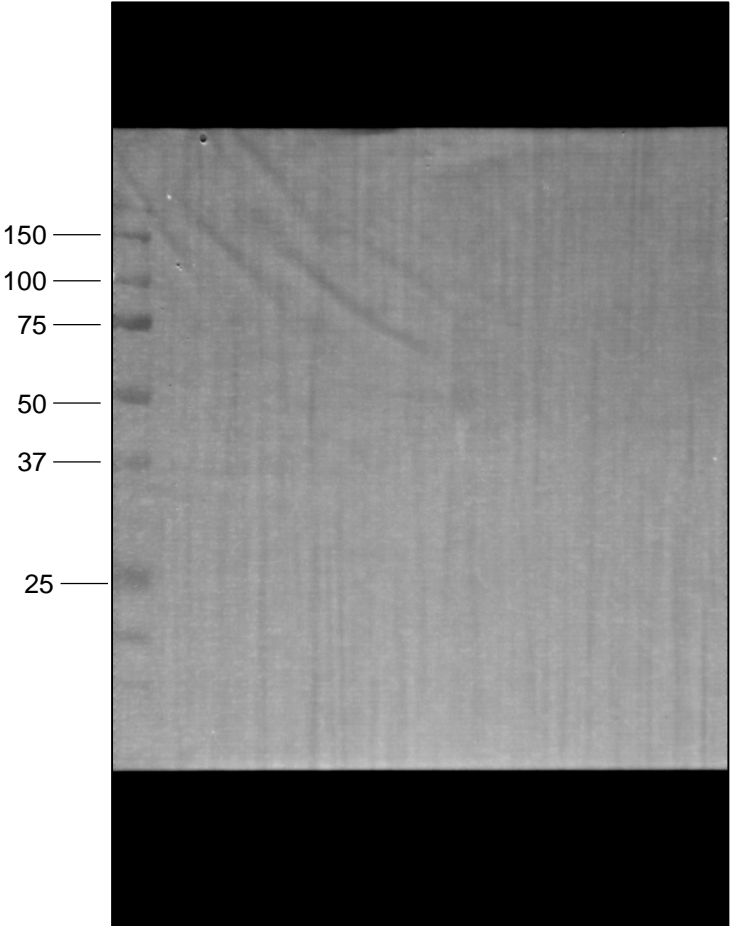

Supplement: SourceData F2 — is the source file for Fig. 2. [file JCB_202301062_SourceDataF2.pdf]

IB; PIGB

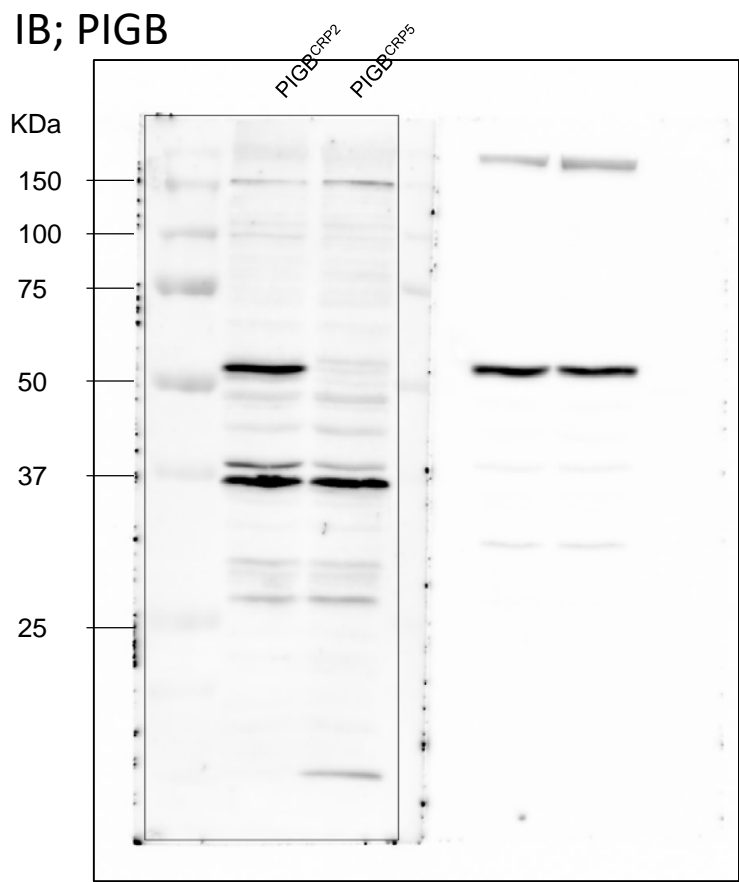

IB;  $\alpha$ -tubulin

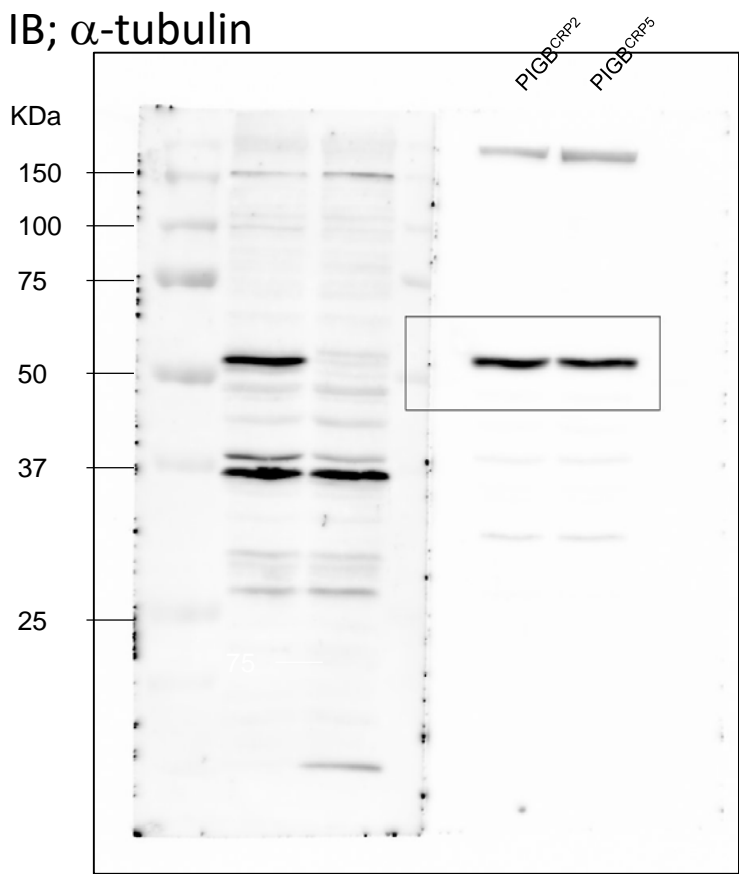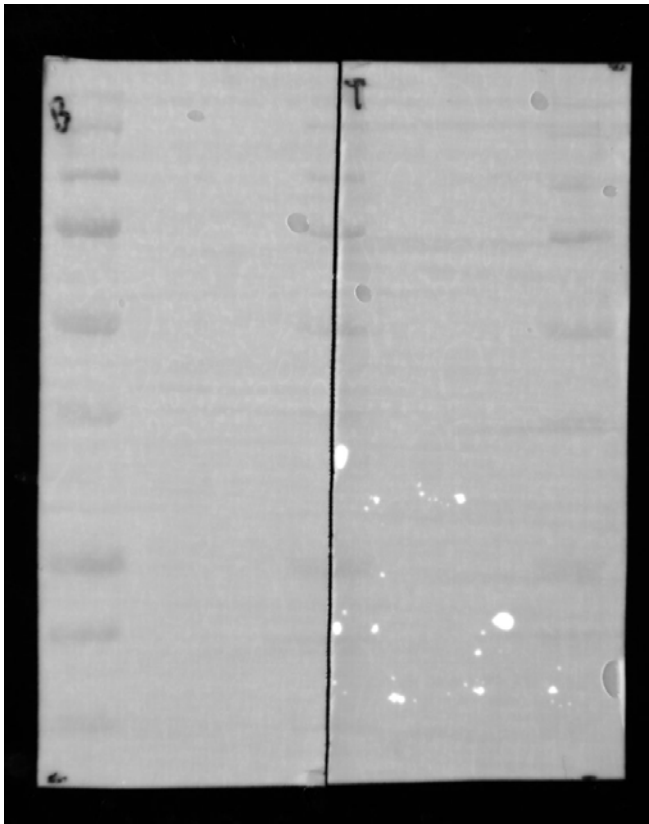

IB; LaminDm0

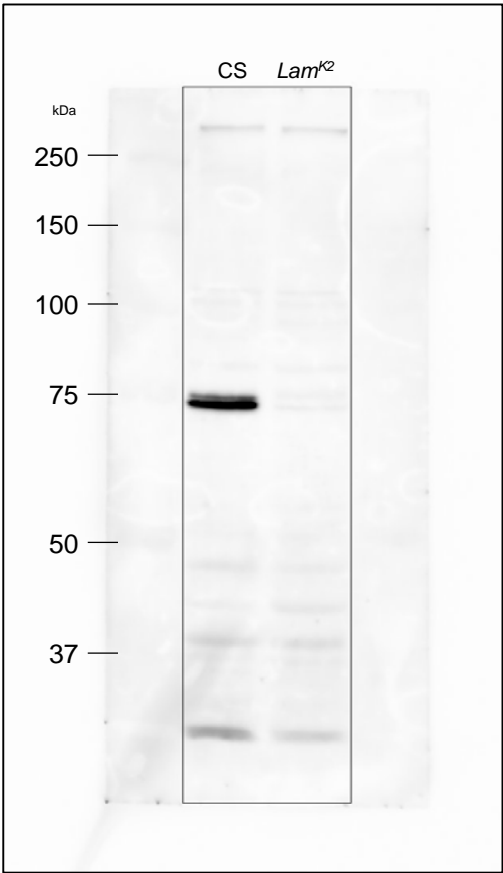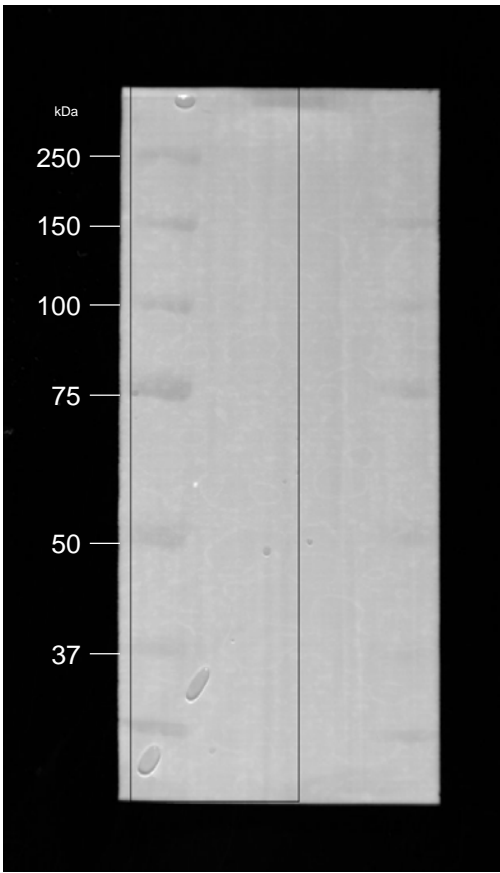

IB;  $\alpha$ -tubulin

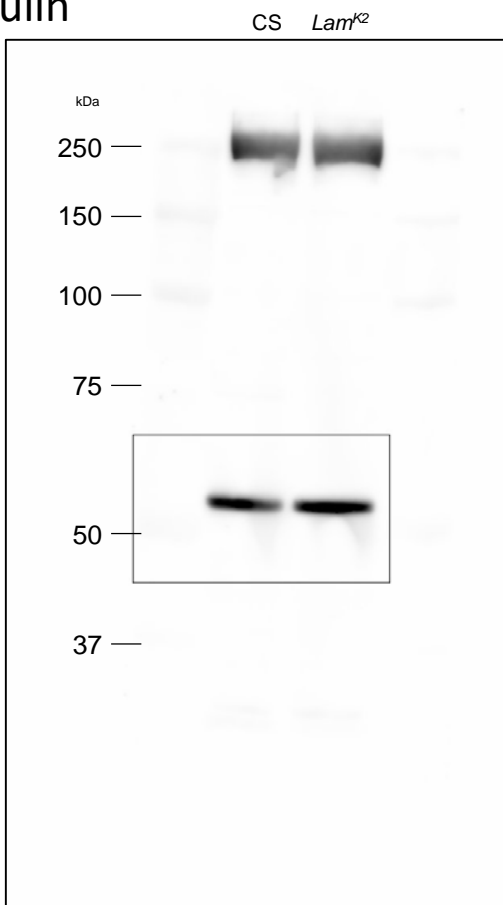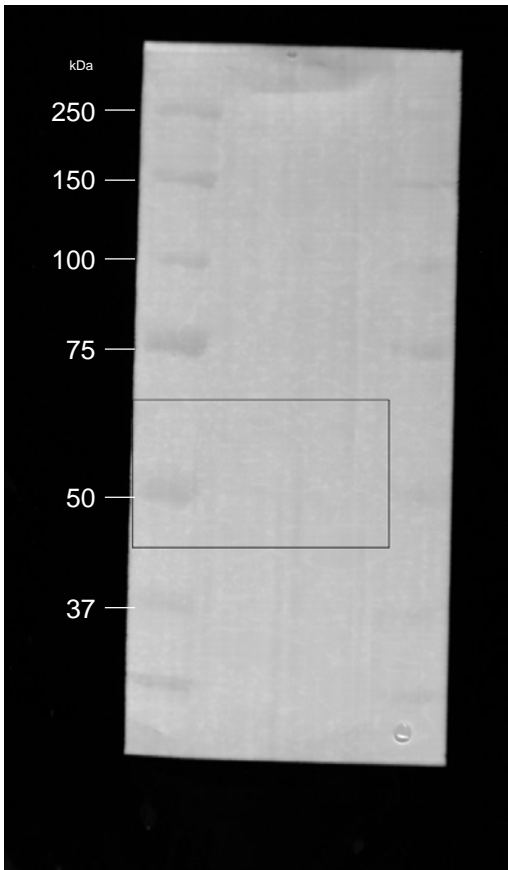

IB; LaminDm0

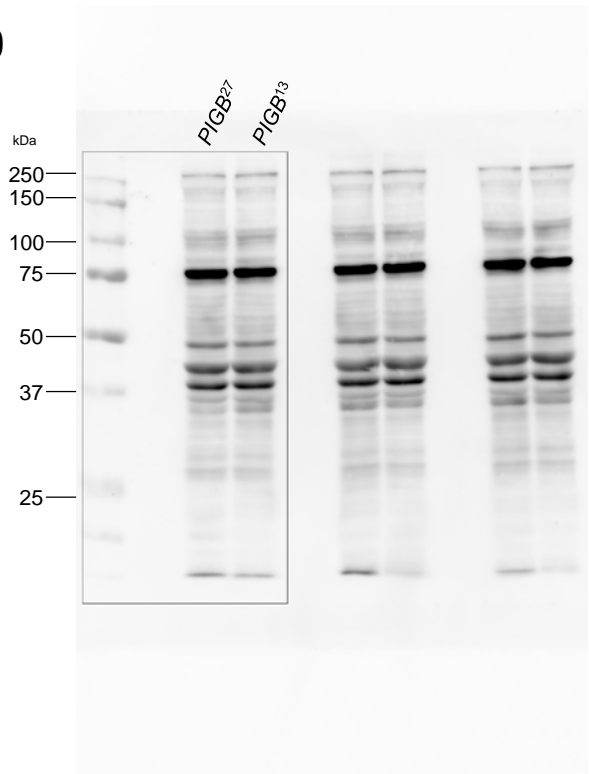

IB; LBR

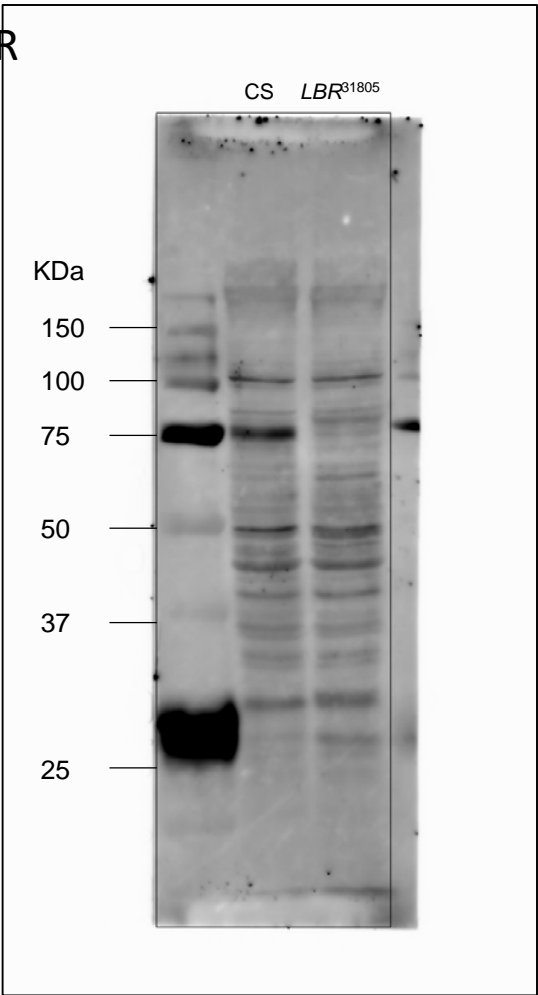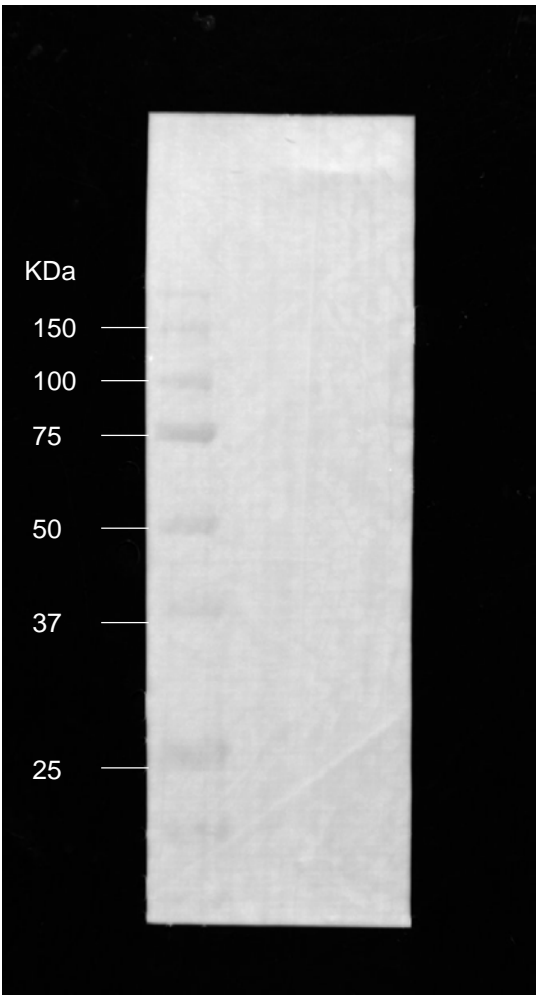

IB;  $\alpha$ -tubulin

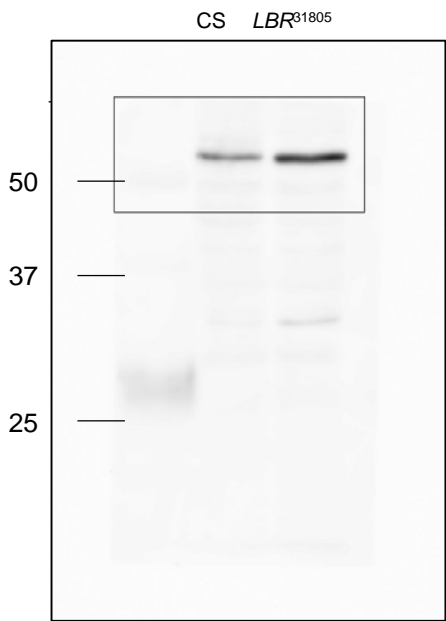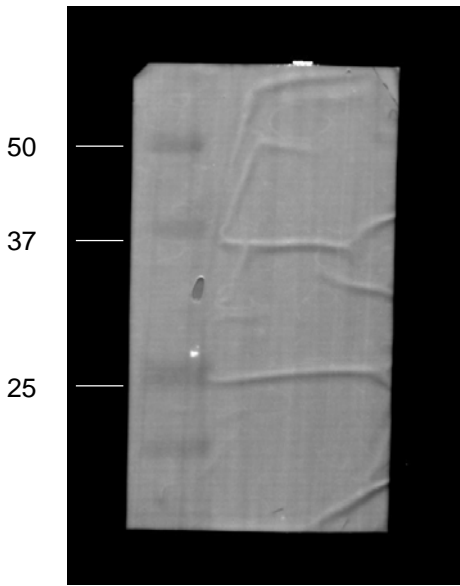

Supplement: SourceData FS1 — is the source file for Fig. S1. [file JCB_202301062_SourceDataFS1.pdf]
