# Supplementary material for: PIGB maintains nuclear lamina organization in skeletal muscle of Drosophila
Source: J Cell Biol. 2024 Jan 23;223(2):e202301062. doi: 10.1083/jcb.202301062 (PMC10808031; doi:10.1083/jcb.202301062)
Supplement: SourceData F4 — is the source file for Fig. 4. [file JCB_202301062_SourceDataF4.pdf]

Source Data; Fig. 4B myc(PIGB) expressed in CHO class B

IB; myc

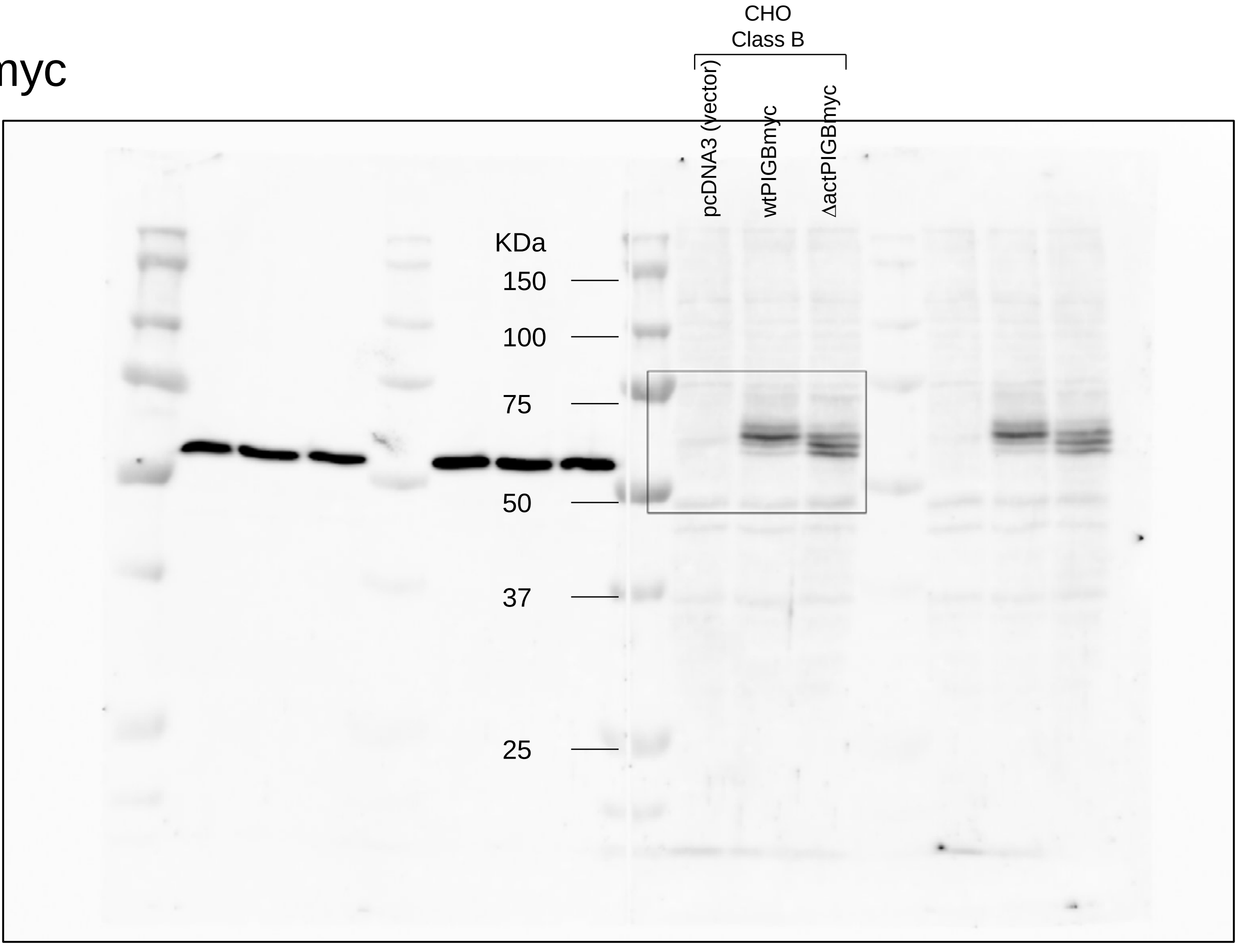

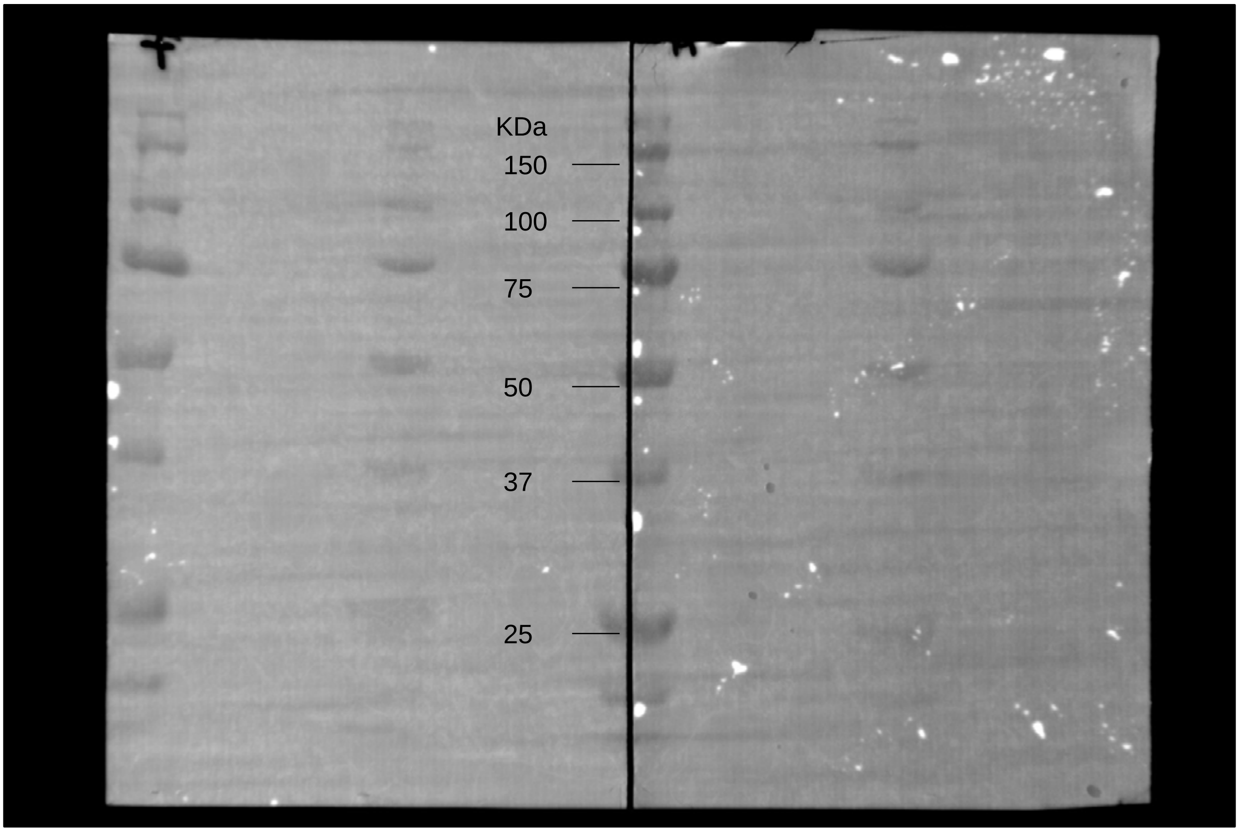

Source Data; Fig. 4B myc(PIGB) expressed in CHO class B

IB; α-tubulin

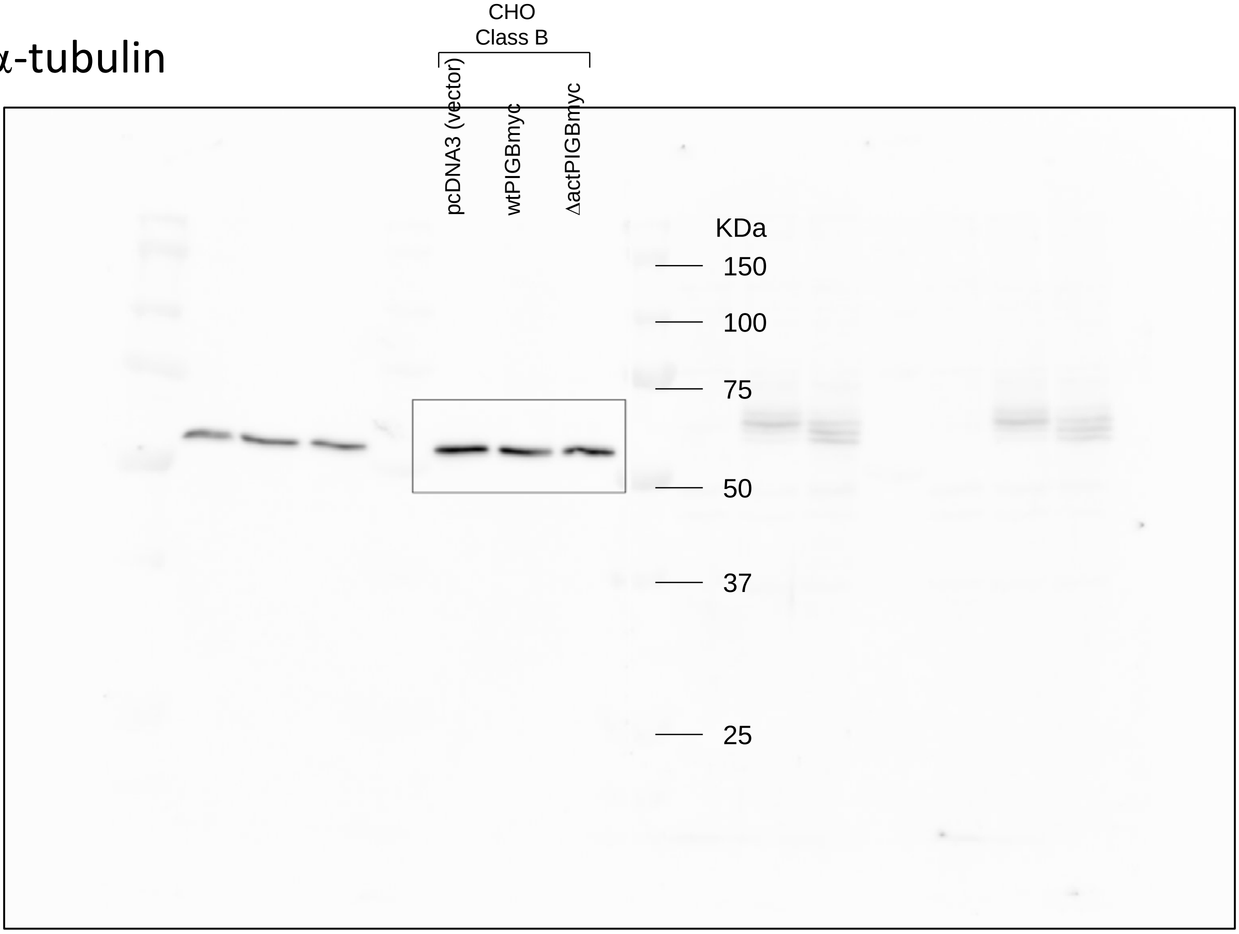

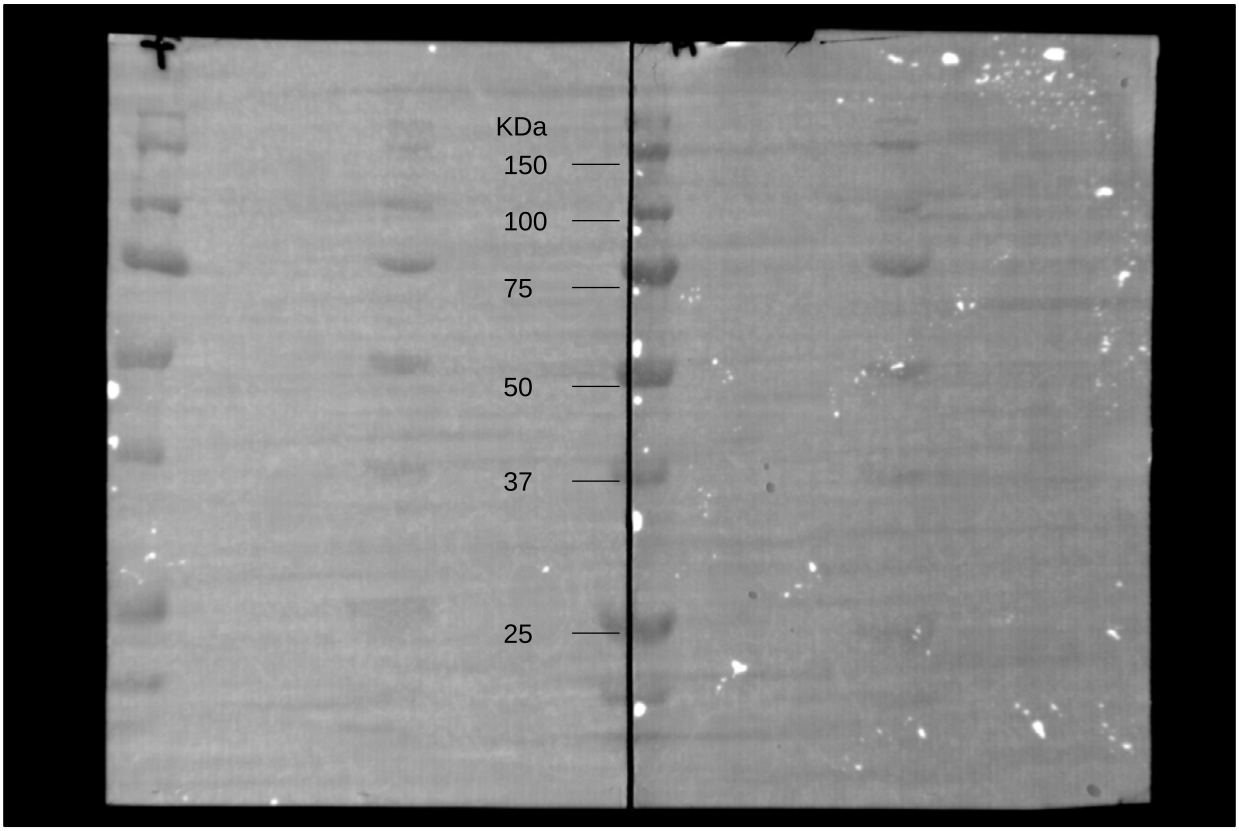

Source Data; Fig. 4B myc(PIGB) expressed in CHO class B for quantification

IB; myc (PIGB)

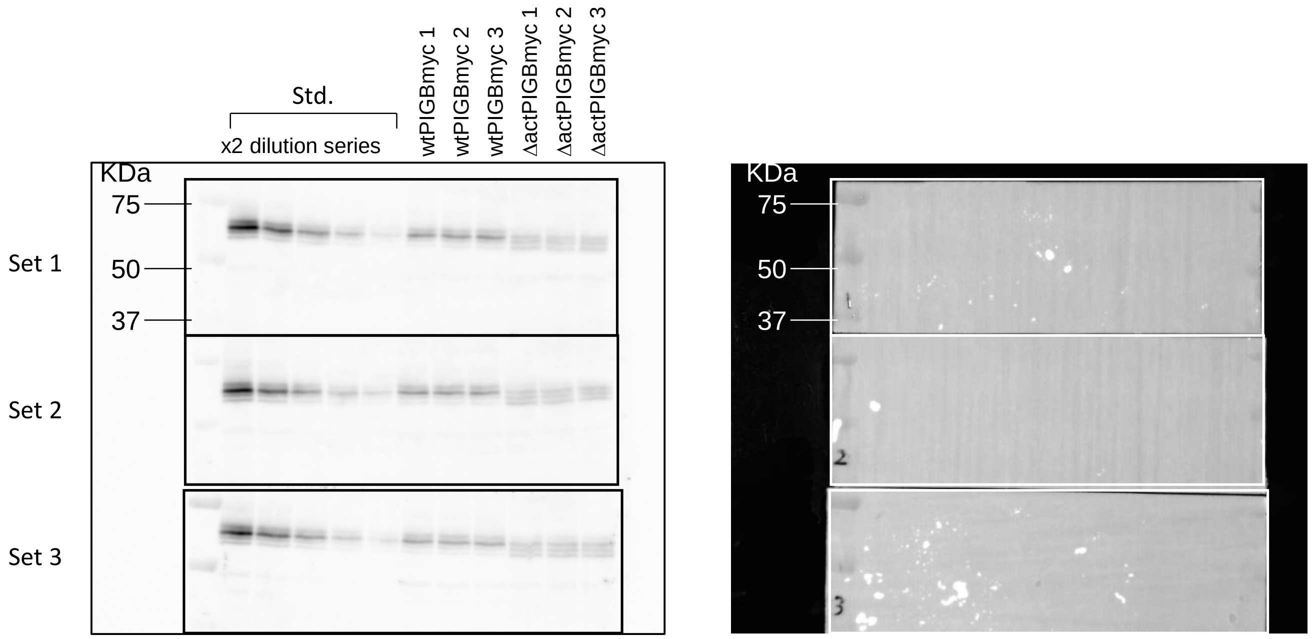

IB; α-tubulin

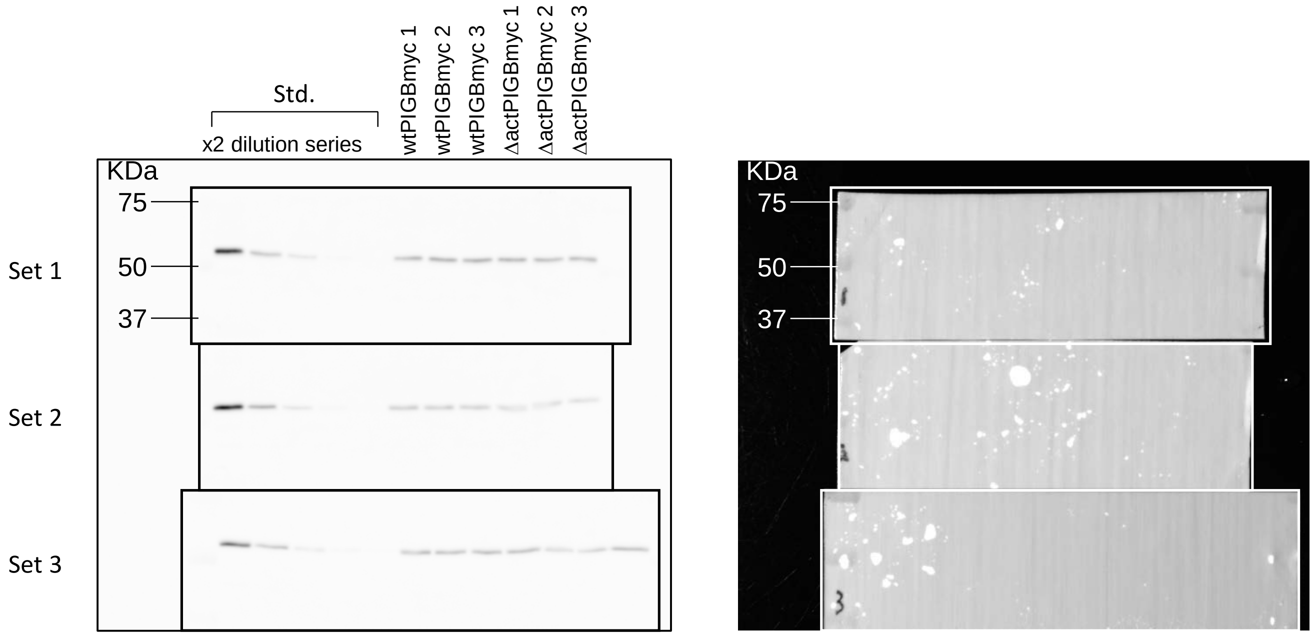

Source Data; Fig.4B myc(PIGB) expressed in S2 cells

IB; myc

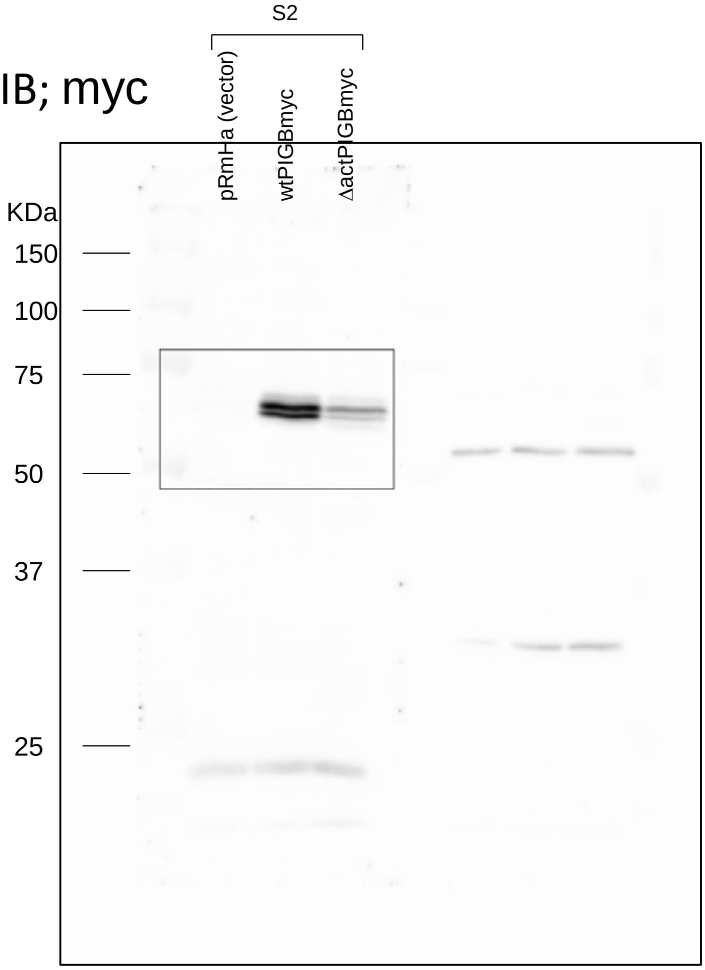

IB; α-tubulin

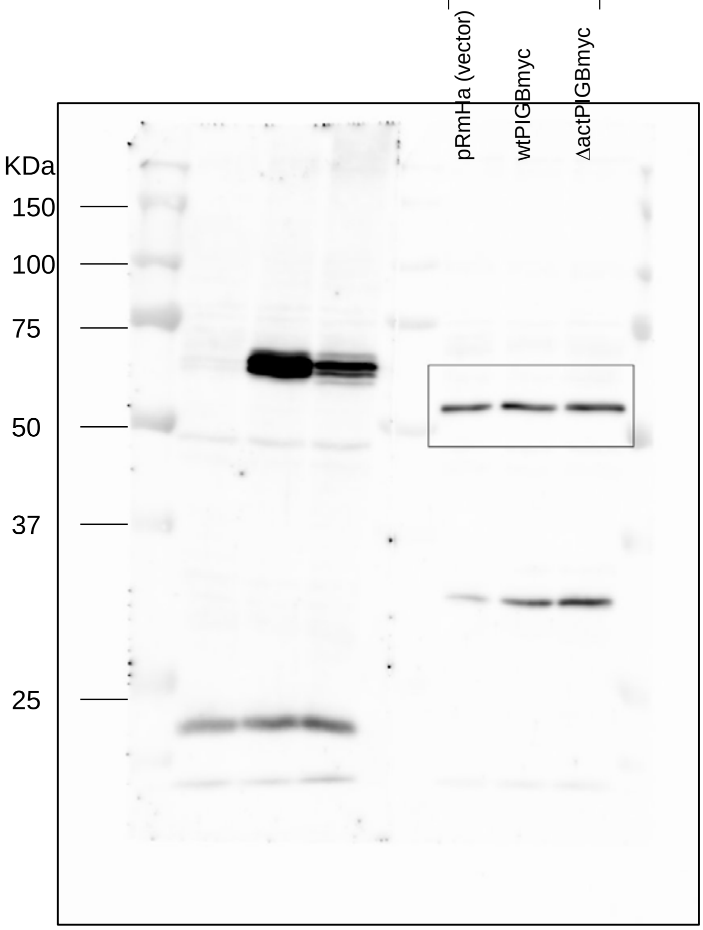

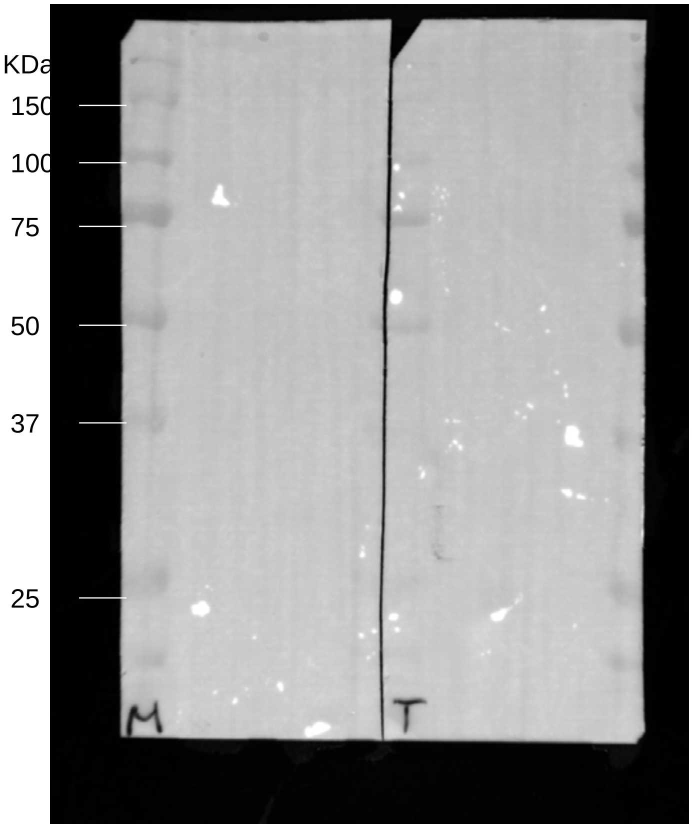

Source Data; Fig. 4B myc(PIGB) expressed S2 cells for quantification

IB; myc (PIGB)

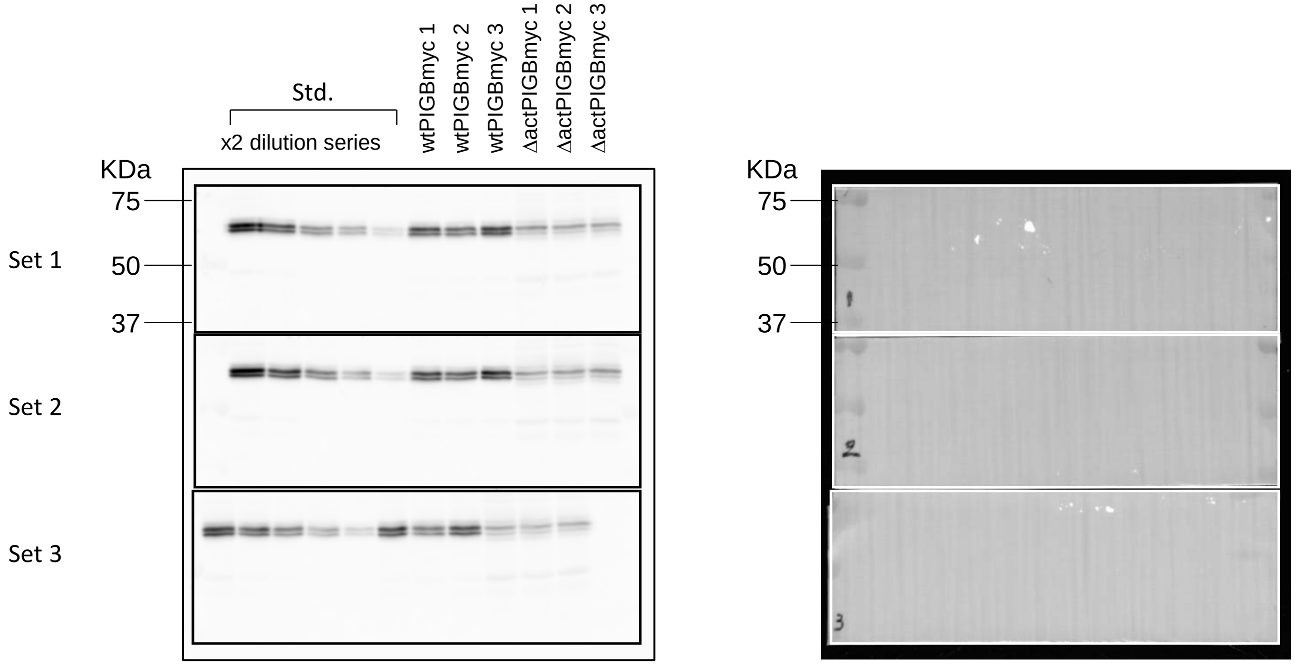

IB; α-tubulin

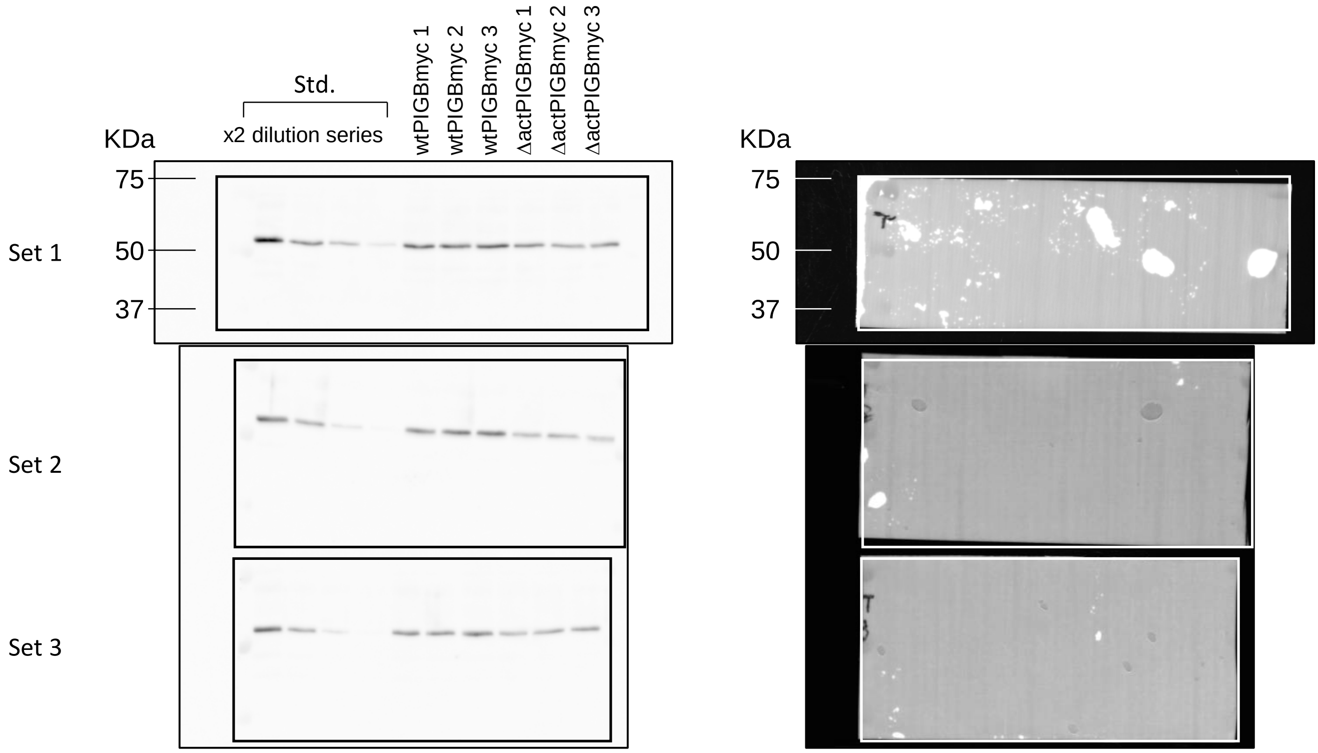

Source Data Fig. 4F

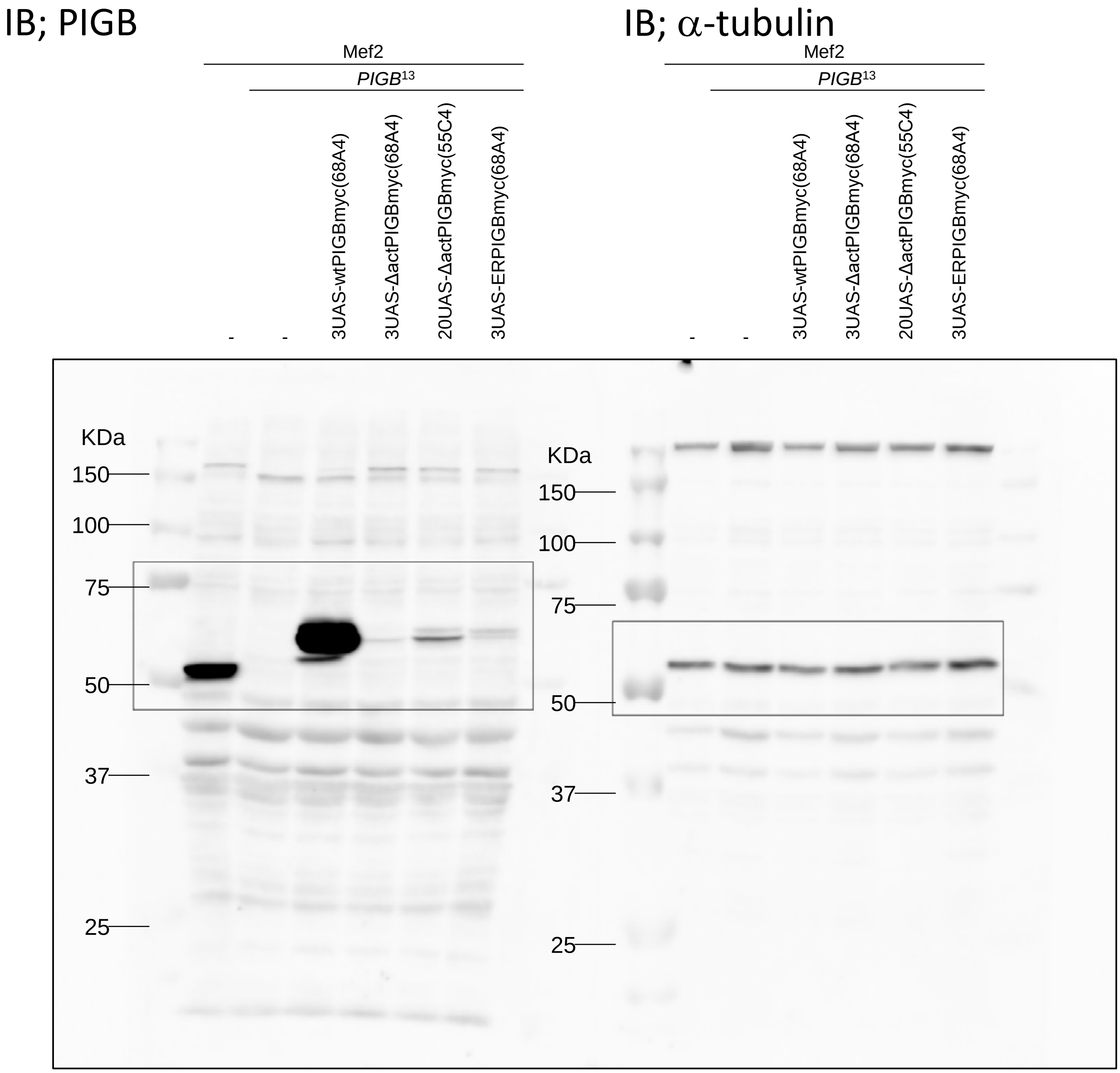

Source Data; Fig. 4G

IB; α-tubulin

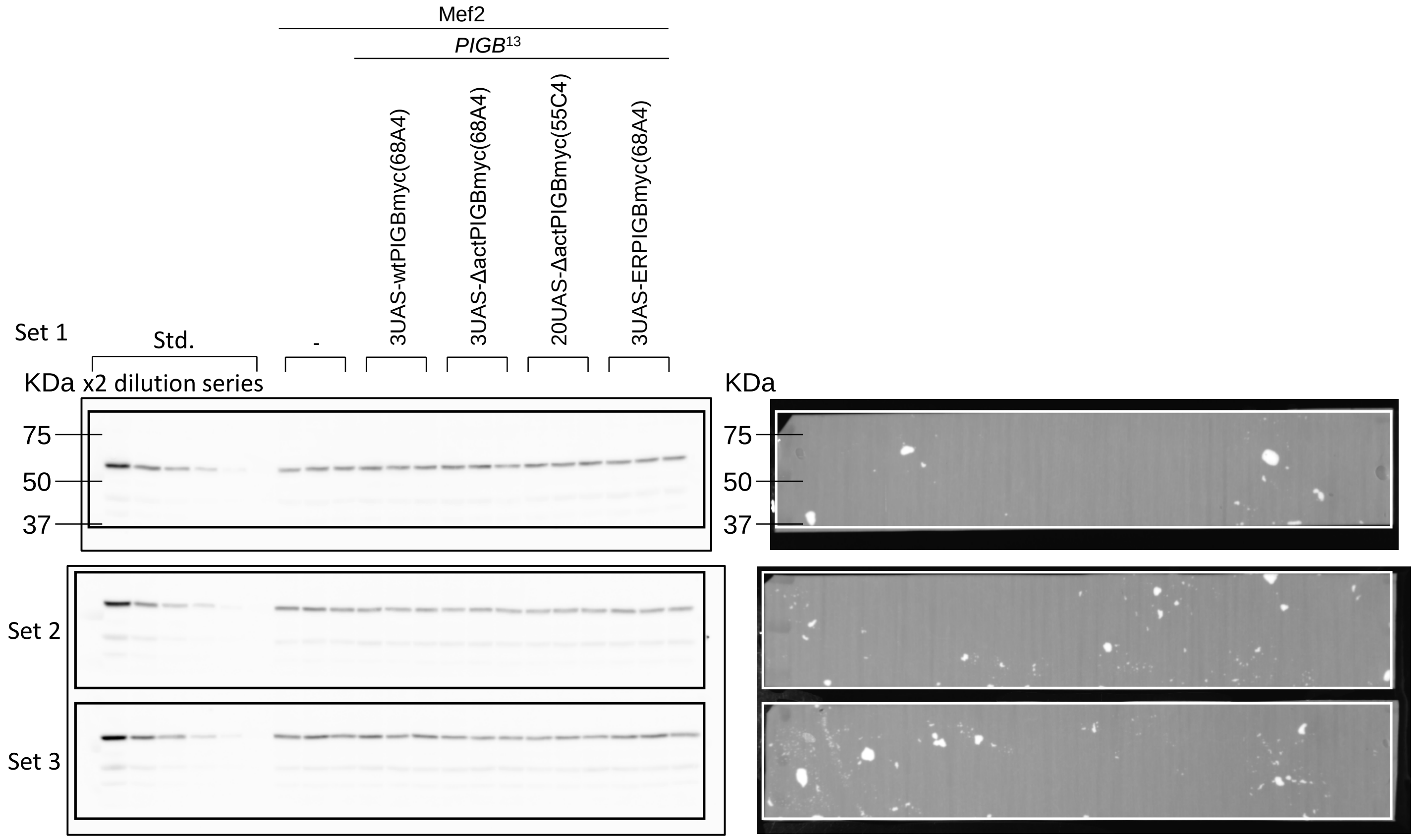

IB; PIGB

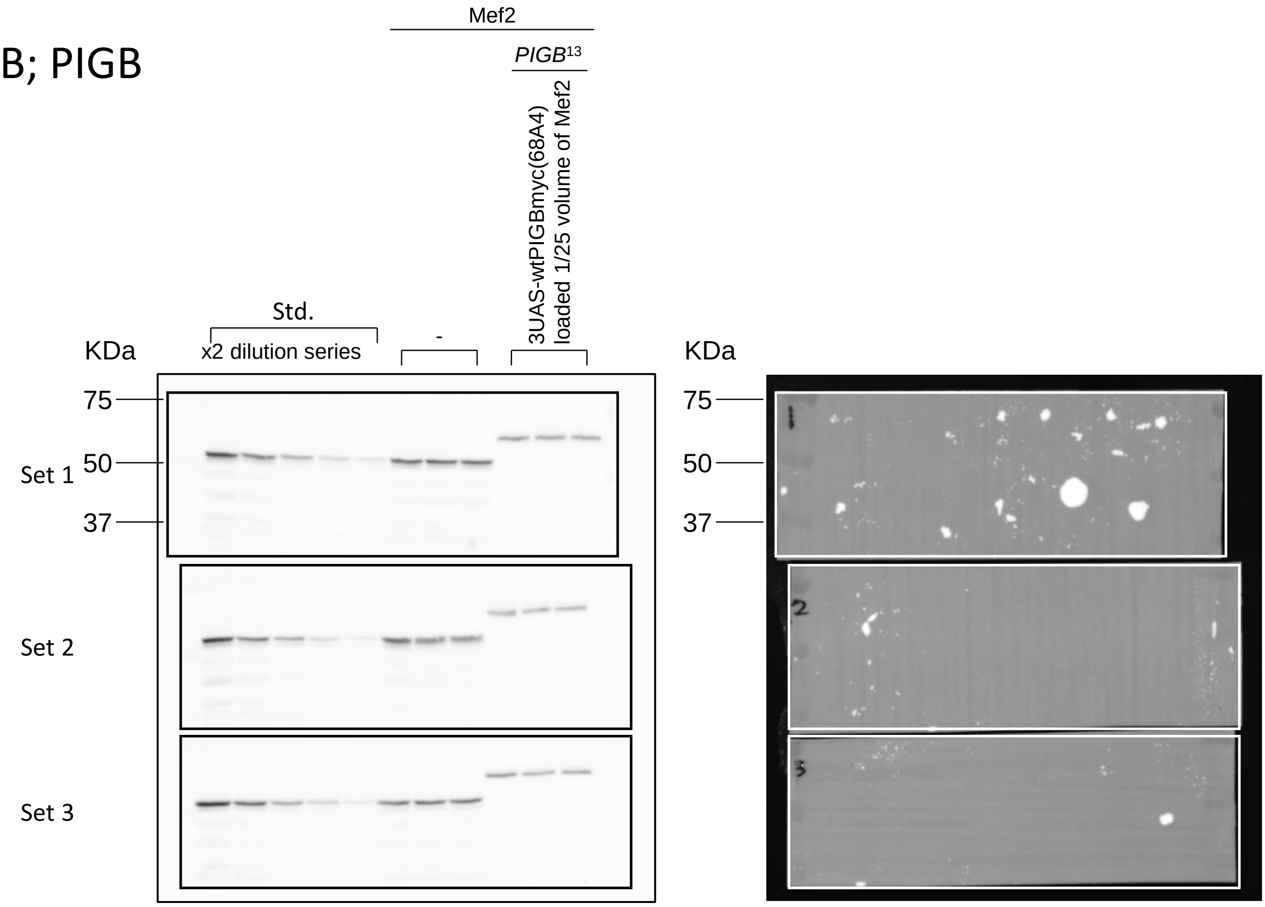

Source Data; Fig. 4G

IB; PIGB

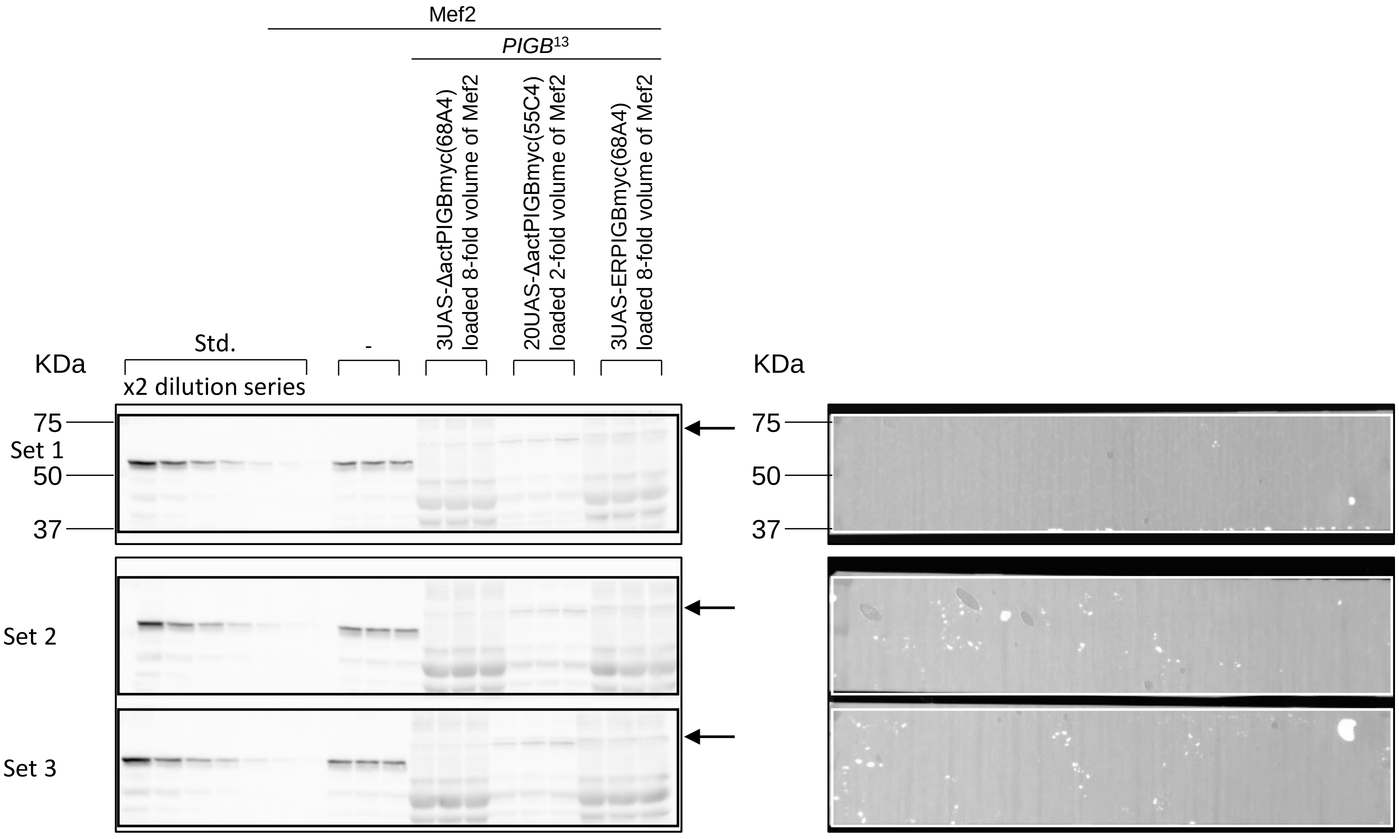

Arrows indicate expressed PIGBmyc variants
